# SRSF1 Is Crucial for Maintaining Satellite Cell Homeostasis During Skeletal Muscle Growth and Regeneration

**DOI:** 10.1002/jcsm.13607

**Published:** 2024-10-09

**Authors:** Zhenzhen Wang, Qian Peng, Zhige Zhang, Xue You, Huimin Duan, Rula Sha, Ningyang Yuan, Zhigang Li, Zhiqin Xie, Jun Han, Ying Feng

**Affiliations:** ^1^ CAS Key Laboratory of Nutrition, Metabolism and Food Safety, Shanghai Institute of Nutrition and Health University of Chinese Academy of Sciences, Chinese Academy of Sciences Shanghai China; ^2^ Department of General Surgery, Zhongshan Hospital Fudan University Shanghai China; ^3^ Lin He's Academician Workstation of New Medicine and Clinical Translation in Jining Medical University, Jining Medical University Jining China

**Keywords:** cellular senescence, dysregulated splicing, muscle regeneration, satellite cells, scRNA‐seq, SRSF1

## Abstract

**Background:**

The splicing factor SRSF1 emerges as a mater regulator of cell proliferation, displaying high expression in actively proliferative satellite cells (SCs). In SRSF1 knockout mice (KO) generated via *MyoD‐Cre*, early mortality and muscle atrophy are observed during postnatal muscle growth. Despite these findings, the precise mechanisms through which SRSF1 loss influences SCs' functions and its role in muscle regeneration remain to be elucidated.

**Methods:**

To unravel the exact mechanisms underlying the impact of SRSF1 deficiency SC functions, we employed single‐cell RNA sequencing (scRNA‐seq) on a mononuclear cell suspension isolated from the newborn diaphragm of KO and control mice. Concurrently, we subjected diaphragm muscles to RNA‐seq analysis to identify dysregulated splicing events associated with SRSF1 deletion. For the analysis of the effect of SRSF1 deletion on muscle regeneration, we generated mice with inducible SC‐specific *Srsf1* ablation through *Pax7‐CreER*. SRSF1 ablation was induced by intraperitoneal injection of tamoxifen. Using cardiotoxin‐induced muscle injury, we examined the consequences of SRSF1 depletion on SC function through HE staining, immunostaining and EdU incorporation assay. C2C12 myoblasts and isolated myoblasts were employed to assess stem cell function and senescence.

**Results:**

Utilizing scRNA‐seq analysis, we observed a noteworthy increase in activated and proliferating myoblasts when SRSF1 was absent. This increase was substantial, with the proportion rising from 28.68% in the control group to 77.06% in the knockout group. However, these myoblasts experienced mitotic abnormalities in the absence of SRSF1, resulting in cell cycle arrest and the onset of cellular senescence. In the knockout mice, the proportion of Pax7+ cells within improper niche positioning increased significantly to 25% compared to 12% in the control cells (*n* ≥ 10, *p* < 0.001). Furthermore, there was an observation of persistent cell cycle exit specifically in the Pax7+ cells deficient in SRSF1 (*n* = 6, *p* < 0.001). SRSF1 plays a pivotal role in regulating the splicing of Fgfr1op2, favouring the full‐length isoform crucial for mitotic spindle organization. Disrupting SRSF1 in C2C12 and primary myoblasts results in multipolar spindle formation (*p* < 0.001) and dysregulated splicing of Fgfr1op2 and triggers cellular senescence. Consequently, adult SCs lacking SRSF1 initially activate upon injury but face substantial challenge in proliferation (*n* = 4, *p* < 0.001), leading to a failure in muscle regeneration.

**Conclusions:**

SRSF1 plays a critical role in SCs by ensuring proper splicing, maintaining mitotic progression and preventing premature senescence. These findings underscore the significant role of SRSF1 in controlling SC proliferation during skeletal muscle growth and regeneration.

## 1 Introduction

Skeletal muscle is pivotal for voluntary movements and respiration. When damaged, adult muscle can fully recover due to muscle stem cells known as satellite cells (SCs) located between the muscle basal lamina and the sarcolemma [1, 2]. These cells have the ability to self‐renew and undergo myogenic differentiation [3]. Pax7, a key marker, plays a vital role in establishing and maintaining the adult SC pool [4].

During the early stages of development, SCs are abundant but dwindle rapidly, making up only 2%–5% of all nuclei in adults [5, 6]. In adult muscles, SCs are dominant, with a temporary halt in the cell cycle. When muscles are injured, these quiescent Pax7+ cells activate to quickly upregulate the myogenic regulatory factor MyoD and re‐enter the cell cycle. Some of the offspring cells, called myoblasts, undergo self‐renewal and return to quiescence to replenish the pool of quiescent satellite cells (QSCs). The remaining myoblasts differentiate and contribute to the formation of new muscle fibres.

Age‐related muscle wasting and dystrophy are tied to declining SC number and their ability to multiply [7, 8, 9]. The ability of SCs to proliferate and return to quiescence is thus crucial for maintaining the stem cell population [10]. Various elements, including SC niche, intrinsic and extrinsic factors have been associated with SC dysfunction. Studies have shown that removing N‐cadherin and M‐cadherin in SCs can activate QSCs without injury [11]. However, reduced fibronectin or loss of integrin β1 hinders self‐renewal and contributes SC ageing [12, 13]. Spry1, a receptor tyrosine kinase Fgf signalling inhibitor, helps restore SCs to dormancy after repair [14]. Notch activation leads to the upregulation of Pax7 and downregulation of MyoD, which in turn promotes the self‐renewal of SCs.

Sarcopenia, a common skeletal muscle disorder, is identified by the progressive atrophy of muscle fibres and contractile dysfunction, accompanied by SC ageing [15]. Ageing SCs display characteristic features such as flattened shape, enlarged nuclei, increased activity of senescence‐associated‐β‐galactosidase (SA‐β‐gal) and higher levels of cyclin‐dependent kinase inhibitors p16^Ink4a^ (p16) or p21^Waf^ (p21), causing irreversible cell cycle arrest [16, 17]. SRSF1, an important regulator of alternative splicing (AS), plays a critical role in maintaining cellular homeostasis across various cell lineages [18, 19, 20, 21, 22]. Remarkably, SRSF1 is highly expressed in actively proliferative SCs. Deleting the *Srsf1* gene in MyoD+ progenitors [23] triggers muscle atrophy, mirroring the observed traits in sarcopenia and age‐related muscle decline [8].

In the present study, we employed scRNA‐seq technology to investigate the detailed mechanisms underlying the impact of SRSF1 loss on SCs' functions. Utilizing scRNA‐seq analysis, we observed a noteworthy increase in activated and proliferating myoblasts when SRSF1 was absent. However, it is worth noting that these myoblasts experienced mitotic abnormalities in the absence of SRSF1, resulting in cell cycle arrest and the onset of cellular senescence. Consistently, SRSF1‐deficient SCs exhibited improper positioning within their niche and entered a permanent state of cell cycle exit. In a consistent fashion, suppressing SRSF1 in myoblasts resulted in the formation of multipolar mitotic spindles and the development of a premature senescent phenotype. Furthermore, our investigation has unveiled that SRSF1 plays a crucial role in facilitating the inclusion of exon4 in Fgfr1op2, resulting in the production of a functional protein. This protein is co‐localized with α‐tubulin during metaphase and is essential for normal spindle organization. Consequently, the absence of SRSF1 in adult SCs renders them incapable of proliferating following injury, resulting in a regeneration failure. Together, these findings strongly indicate that SRSF1 plays a distinct role in governing SC proliferation during skeletal muscle growth and regeneration.

## 2 Methods

### 2.1 Generation of *Srsf1* ^*flox/flox*^;*Pax7‐CreER* Mice, Muscle Injury and Regeneration

The wild‐type (WT) (*Srsf1* ^*flox/flox*^) and knockout (KO) (*Srsf1* ^*flox/flox*^ *;MyoD‐Cre*) mice were previously described [23]. For muscle regeneration studies, *Srsf1* ^*flox/flox*^ were crossed with *Pax7‐CreER* mice [24] to generate mice. These mice were administered tamoxifen (2 mg daily for 5 days) intraperitoneally to induce the deletion of *Srsf1* in SCs. The control group received daily injections of corn oil. Muscle injury and regeneration were assessed in 2‐month‐old mice by injecting 50 μL of cardiotoxin into the tibialis anterior (TA) muscle after disinfection with 75% alcohol. The TA muscles were collected at different time point post‐injury for regeneration analysis. Meanwhile, *Srsf1* ^*flox/flox*^ mice were also injected with tamoxifen and subjected to induced muscle damage to examine the effects of tamoxifen on the muscle regeneration. All experiments were conducted following the guidelines of the Institutional Animal Care and Use Committee of Shanghai Institute of Nutrition and Health, Chinese Academy of Sciences. The primer sequences used in the genotyping can be found in Table S1.

### 2.2 Isolation of Single Cells and scRNA Sequencing

P1 WT and KO mouse diaphragms were minced and enzymatically digested to obtain single cells using dispase II, collagenase type XI and penicillin/streptomycin. After filtration and centrifugation, cells were resuspended in a 20% FBS/F‐10 buffer and processed on a Chromium Single Cell 3′ Chip (10× Genomics). Library sequencing utilized Illumina NovaSeq 6000, and resulting datasets are available in the GEO database under accession number GSE233227.

### 2.3 scRNA‐seq Data Preprocessing

We processed raw data using Cell Ranger software (Version 5.0.0) from 10× Genomics to create a gene count versus cells matrix, following established methods [25]. After quality control, 9499 WT and 5672 KO single cells were retained. Seurat's NormalizeData function normalized library size, and gene expression was log‐transformed using ‘LogNormalize’. Top variable genes across cells were found with the FindVariableGenes function, and PCA reduced dimensionality using RunPCA. Gene expression‐based clustering utilized the FindCluster function, visualized with t‐SNE via RunTSNE. Marker genes for each cluster were found using FindAllMarkers. Differentially expressed genes (DEGs) were identified with FindMarkers (test.use = MAST) using a threshold of *p* < 0.05 and |log2foldchange| > 0.58 in Seurat. GO and KEGG pathway enrichment analysis of DEGs were performed using https://www.bioinformatics.com.cn.

### 2.4 Pseudotime Trajectory Analysis and RNA Velocity Analysis

Monocle2 (Version 2.9.0) organized SKM cells in ‘pseudotime’ and positioned them along an inferred trajectory [26]. Seurat's raw data were converted to the CellDataSet object using Monocle2's importCDS function. Genes for cell ordering were identified via differentialGeneTest. Clustering used reduceDimension, and trajectory was inferred with orderCells. Gene expression changes along pseudotime trajectory were observed using plot_genes_in_pseudotime. Based on the output of Cell Ranger, the spliced and unspliced reads were calculated separately using the Python script velocyto.py [27]. The RNA velocities for each gene in each cell were then calculated using the Python package scVelo [28] and projected onto a t‐SNE plot for presentation.

### 2.5 Statistical Analyses

Statistical analysis was conducted using GraphPad Prism 8.0 software. Histograms display the mean ± standard deviation (SD) and were obtained from a minimum of three separate experiments. For multiple experimental groups, one‐way or two‐way ANOVA analysis followed by Tukey's or Dunnett's test was employed. For comparison between two experimental groups, the two‐tailed unpaired Student's *t*‐test was used. Representative western blot results were shown from three independent experiments. The protein quantification analysis was performed using ImageJ. Statistical significance was defined as *p < 0.05, **p < 0.01 and ***p < 0.001.

## 3 Results

### 3.1 scRNA‐seq Showed a Reduced Count of Skeletal Muscle Cells With an Irregular Distribution in the KO Diaphragm

**FIGURE 1:**
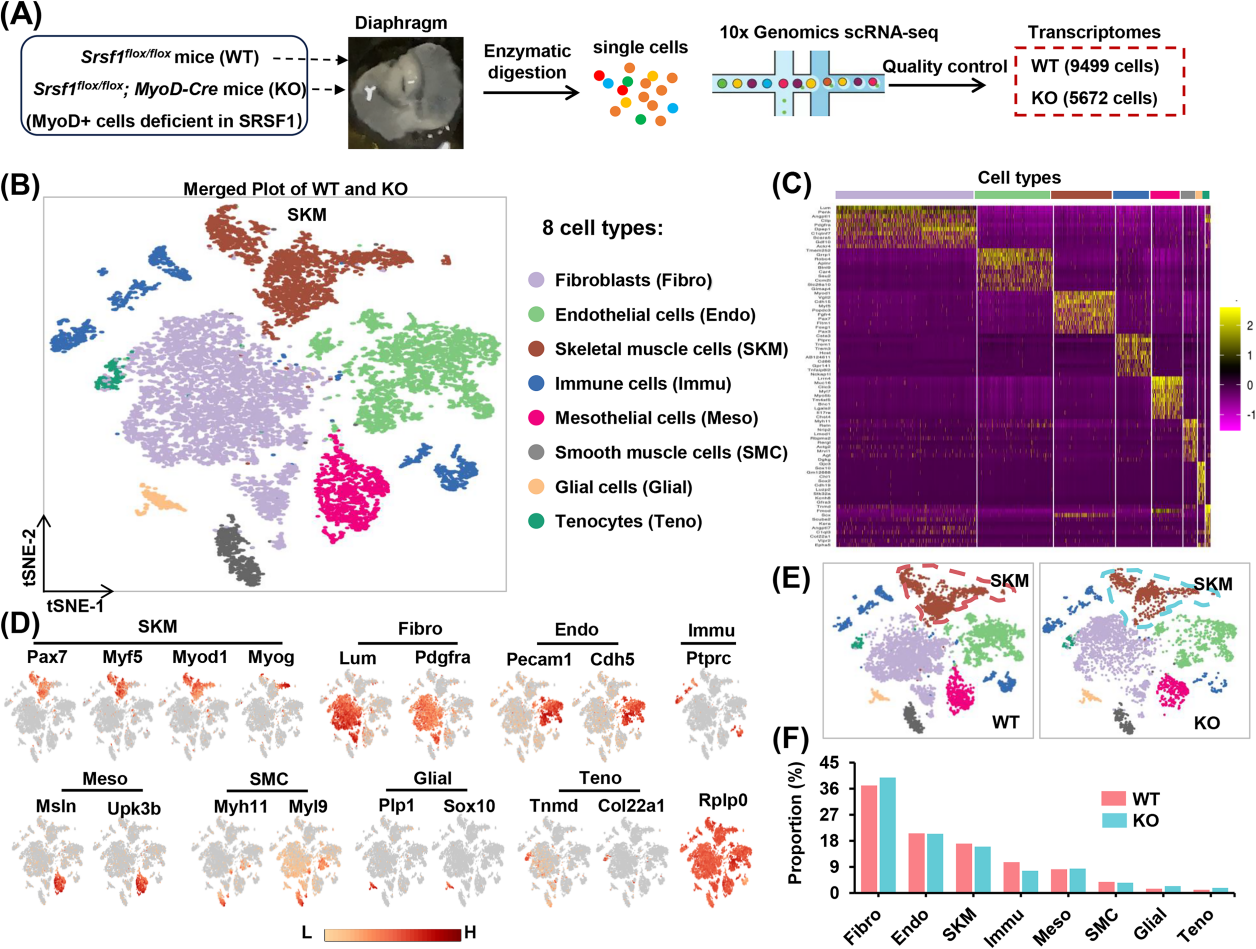
scRNA‐seq showed a reduced number of skeletal muscle cells with abnormal distribution in the KO diaphragm. (A) The process involved diaphragm preparation from *Srsf1* ^*flox/flox*^ (WT) and *Srsf1* ^*flox/flox*^ *; MyoD‐Cre* (KO) mice immediately after birth, single‐cell isolation and the construction of chromium 10× Genomics library for scRNA‐seq analysis. (B) The t‐SNE plot was employed to depict eight distinct cell types within diaphragm muscles, colour‐coded for identification. (C) A heatmap was generated to display the top 10 marker genes for each identified cell type. (D) Specific cell marker gene expression was visualized through t‐SNE plots to distinguish the eight cell types. (E) Individual t‐SNE plots illustrated the distribution of SKM and other cell types in both WT and KO groups. (F) Comparisons were made between the two groups to determine the proportion of each cell type present.

Previous research revealed that the absence of SRSF1 in MyoD+ progenitors significantly impaired SC proliferation and caused muscle atrophy [23]. For deeper insights into the impact of SRSF1 loss on SC functions, we performed scRNA‐seq on diaphragm mononuclear cells from WT *(Srsf1* ^*flox/flox*^ *)* and KO *(Srsf1* ^*flox/flox*^ *; MyoD‐Cre)* mice right after birth (Figure 1A). After quality control (Figure S1), we analysed 15 171 transcriptomes, comprising 9499 control cells and 5672 KO cells (Figure 1A).

Utilizing t‐distributed stochastic neighbour embedding (t‐SNE) plots, we classified eight distinct cell types (Figure 1B), each characterized by unique transcriptional programs and the expression of lineage‐specific markers (Figure 1C,D). Notably, skeletal muscle cells (SKM) expressed myogenic lineage markers like *Pax7*, *Myf5*, *Myod1* and *Myog*. Fibroblasts (Fibro) were enriched for *Lum* and *Pdgfra*, whereas endothelial cells (Endo) highly expressed *Pecam1* and *Cdh5*. Immune cells (Immu) specifically expressed *Ptprc*, mesothelial cells (Meso) were enriched for *Msln* and *Upk3b*, and smooth muscle cells (SMC) exhibited high expression of *Myh11* and *Myl9*. Glial cells were enriched for *Plp1* and *Sox10*, and tenocytes (Teno) expressed *Tnmd* and *Col22a1*. The housekeeping gene *Rplp0* was expressed across all cell populations.

Individual t‐SNE plots unveiled notable changes in the distribution of SKM cells within the KO diaphragm when compared to the control, indicating a shift in cell fate as a response to the absence of SRSF1 (Figure 1E). Additionally, these alterations were accompanied a reduction in the SKM population and an increase in the Fibro population in the KO group (Figure 1F).

### 3.2 The Absence of SRSF1 Hampered Both the Self‐Renewal and Differentiation Abilities of Myoblasts

**FIGURE 2:**
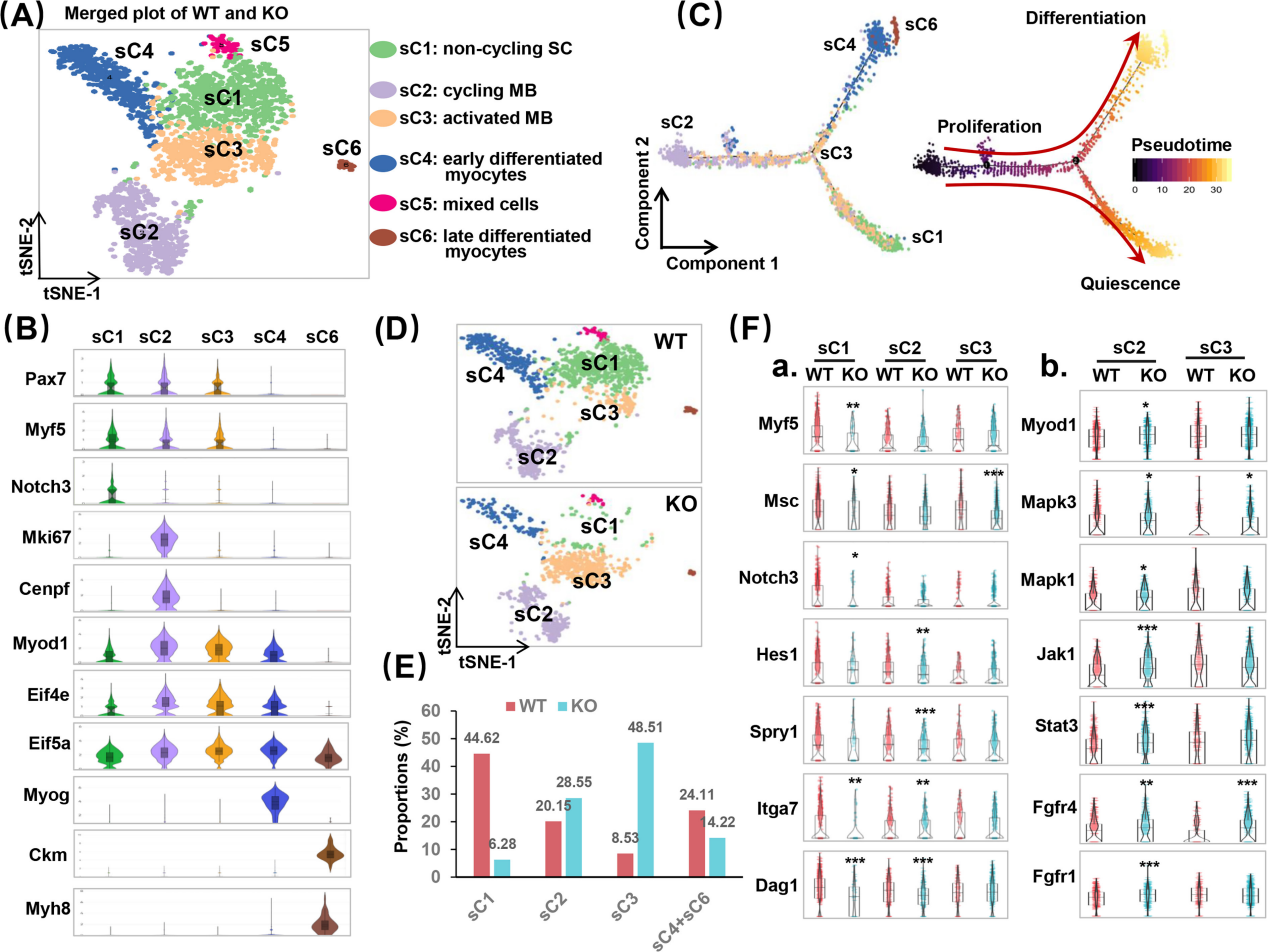
The absence of SRSF1 led to an accumulation of activated and cycling myoblasts while impairing myogenic differentiation. (A) A t‐SNE plot displayed six distinct subpopulations of combined WT and KO skeletal muscle (SKM) cells. (B) A violin plot illustrated marker gene expression in the different subclusters. (C) The trajectory plot of SKM cells was colour‐coded by subclusters on the left and pseudotime order on the right. The trajectory was divided into two branches, indicating two distinct cell fates. (D) t‐SNE plots showed WT SKM subclusters in the top panel and KO SKM subclusters in the bottom panel. Different colours represented different subclusters as described in (A). (E) The proportion of non‐cycling MB (sC1), cycling MB (sC2), activated MB (sC3) and differentiated myocytes (sC4 and sC6) was compared between the two groups. (F‐a‐b) Violin plots showed the expression levels of specific genes between WT and KO subclusters.

We then conducted unsupervised cluster analysis on the SKM population, leading to identification of six subclusters with distinct transcriptomes (sC1 to sC6) (Figures 2A and S2A). As illustrated in Figure 2B, three of these subclusters (sC1, sC2 and sC3) exhibited similar expression patterns of SC markers *Pax7* and *Myf5*. Notably, sC1 cells were enriched for QSC markers such as *Notch3* and displayed low levels of *Myod1*. sC1 cells were thus identified as non‐cycling SCs. sC2 cells showed high levels of proliferation markers *Mki67* and *Cenpf*, indicating that they were actively cycling myoblasts (MB). sC3 cells were defined as activated MB, expressing high levels of SC activation markers *Myod1* and translation‐related genes *Eif4e* and *Eif5a*, similar to sC2 cells. They did not express *Mki67* compared to sC2 cells or *Notch3* compared to sC1 cells (Figure 2B). Differential gene expression analysis confirmed that sC3 cells were distinct from sC1 and sC2 cells (Figure S3). Additionally, sC4 cells and sC6 cells were classified as early differentiated myocytes and late differentiated myocytes, respectively, based on the expression of differentiation markers such as *Myog*, *Ckm*, *Myh8*, *Myot and Tmod4* (Figures 2A and S2B). sC5 cells exhibited a mixed gene expression profile and were excluded from further analysis.

We then used Monocle2 [26] for the analysis of dynamic cell transitions during perinatal myogenesis, which resulted in the organization of the five subclusters into two major trajectories (Figure 2C). Notably, sC2 cells were positioned at the beginning of pseudotime trajectory, whereas sC4 and sC6 cells were situated at the terminal points of differentiation branch. In contrast, sC1 cells were positioned along the quiescence branch, and sC3 cells were dispersed along the junction of two branches. These findings suggest that the cycling myoblasts during perinatal myogenesis can choose between differentiation and entering into a quiescence state. We also performed RNA velocity analysis, which showed strong directional flows toward two main branches: early differentiated MB (blue arrow) and non‐cycling SCs (green arrow) (Figure S2C). Interestingly, most RNA velocity vectors within the cycling MB pointed in the opposite direction (red curved arrow), indicating that these cells are actively cycling and will eventually exit the cell cycle for either differentiation or quiescence.

Upon examining individual t‐SNE plots (Figure 2D) and comparing the cell numbers and proportions of each subcluster between the WT and KO groups (Figure 2E), noteworthy differences emerged. In the WT group, non‐cycling SCs comprised 44.62% of the SKM population, whereas cycling and activated myoblasts (sC2 and sC3) accounted for 20.15% and 8.54%, respectively. In stark contrast, the deficiency of SRSF1 resulted in a significant increase in the population of cycling and activated myoblasts (28.55% and 48.51%), accompanied by a substantial decrease in non‐cycling SCs (6.28%). Concomitantly, the proportion of differentiated myocytes (sC4 and sC6) decreased from 24.11% in the WT group to 14.22% in the KO group. In the KO group, changes in subcluster distribution correlate with diminished expression of SC markers (*Myf5* and *Msc*), quiescence‐related genes (*Notch3*, *Hes1* and *Spry1*) and niche components (*Itga7* and *Dag1*) in sC1 and sC2, but not in sC3 except for *Msc* (Figure 2F‐a). This aligned with higher Myod1 levels and increased expression of cell proliferation pathways (*Mapk1*, *Jak/Stat3* and *Fgf*) in sC2 or sC3 (Figure 2F‐b). Additionally, there was significant upregulation in MAPK‐related genes (*Mapk1*, *Mapk3* and *Map 2k3*) and dysregulation in genes linked to skeletal muscle differentiation (*Myog*, *Prox1*, *Vgll* and *Rb1*) in the sC4 cells of the KO group (Figure S4). These findings strongly indicated that the lack of SRSF1 compromised both the self‐ renewal and differentiation abilities of myoblasts.

### 3.3 The Absence of SRSF1 Induces Mitotic Abnormalities, Cell Cycle Arrest and Cellular Stress

**FIGURE 3:**
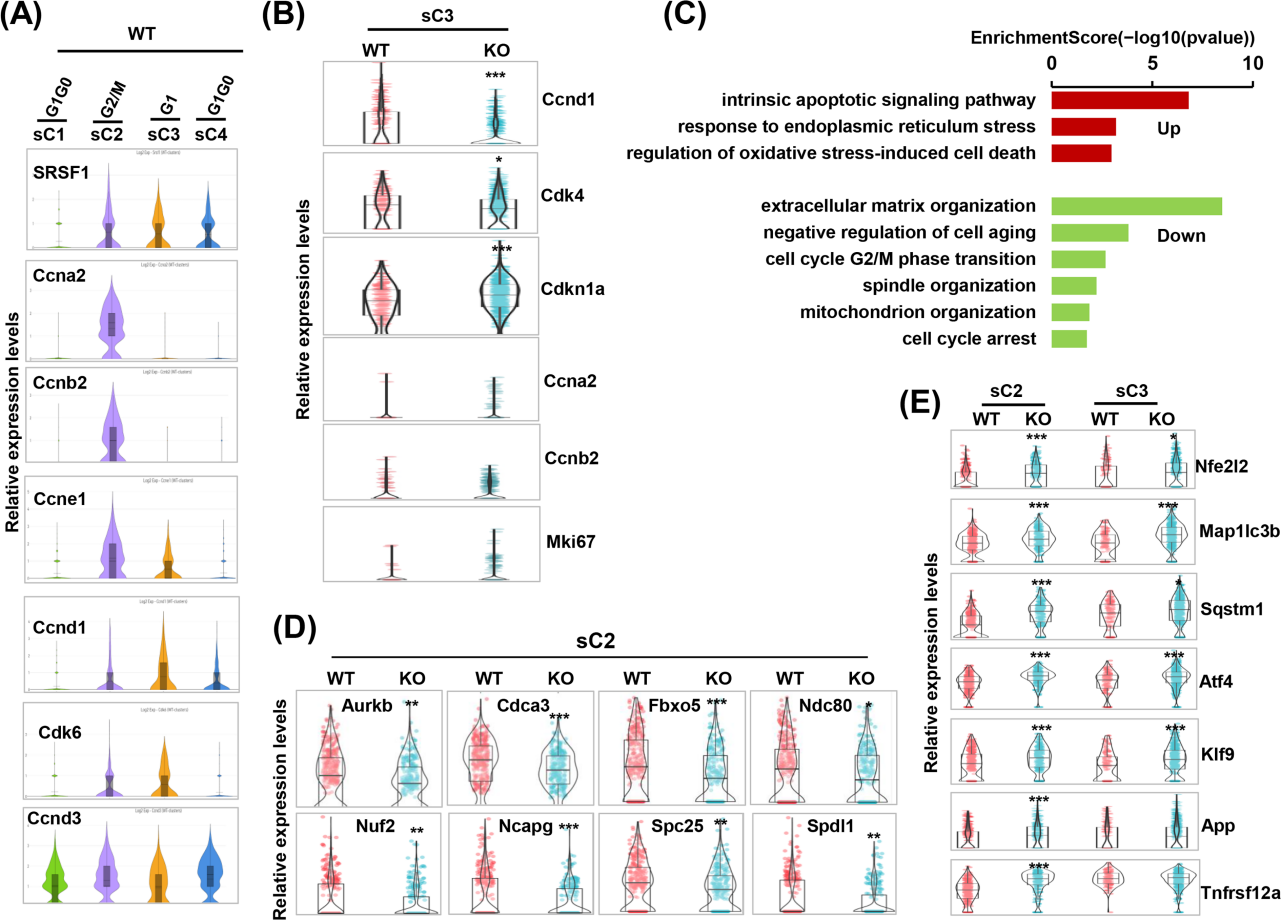
Deletion of SRSF1 induces mitotic abnormalities in cycling myoblasts, leading to cell cycle arrest and cellular senescence. (A) Violin plots illustrate the expression of cell cycle–related genes and SRSF1 in WT subclusters. (B) Violin plots display the expression levels of cell cycle–related genes in activated MB (sC3) between the WT and KO groups. (C) GO enrichment analysis was conducted to identify upregulated and downregulated terms in cycling MB (sC2) of the KO group compared to the control. (D) Violin plots show the expression levels of genes involved in mitotic spindle organization and cell division in cycling MB (sC2) between the WT and KO groups. (E) Violin plots displayed the expression levels of indicated genes involved in cell death and stress in activated MB (sC3) and cycling MB (sC2) between the WT and KO groups.

Examination of cell cycle–related gene expression across SKM subclusters of the WT group revealed that the sC2 cells exhibited heightened expression of G2/M‐cyclins (*Ccna2*, *Ccnb2* and *Ccne1*) (Figure 3A), indicating progression through the mitosis. Conversely, sC3 cells showed elevated levels of G1‐cyclin and kinase (*Ccnd1* and *Cdk6*), indicating their presence in the G1 phase. SRSF1 expression was notably present in sC2 and sC3 myoblasts, closely tied to cell cycle progression. However, sC4 exhibited low SRSF1 expression, whereas sC1 had nearly absent SRSF1, in line with low levels of *Ccnd1* and *Cdk6*, indicative of their exit from the cell cycle.

Compared to the sC3 of the WT group, differential gene expression analysis revealed decreased levels of *Ccnd1* and *Cdk4*, along with increased expression of the cell cycle inhibitor *Cdkn1a* in the sC3 of the KO group (Figure 3B). Given the increased accumulation of sC3 cells in the KO group (Figure 2D,E), coupled with low levels of *Ccna2*, *Ccnb2* or *Mki67* expression, it can be inferred that sC3 cells of the KO group were arrested in the G1 phase. Moreover, GO enrichment analysis unveiled downregulation of terms related to cell cycle G2/M phase transition and spindle organization in sC2 cells of the KO group (Figure 3C). Conversely, the intrinsic apoptotic signalling pathway was among the most significantly upregulated terms in KO myoblasts. Notably, sC2 cells in the KO group displayed substantial downregulation of genes responsible for spindle formation and mitotic progression, such as *Aurkb*, *Cdca3*, *Fbxo5*, *Ndc80*, *Nuf2*, *Ncapg*, *Spc25* and *Spdl1* (Figure 3D). In contrast, genes related to autophagy, oxidative stress and inflammation *(Nfe2l2*, *Map 1lc3b*, *Sqstm1*, *Atf4*, *Klf9*, *App* and *Tnfrsf12a)* were upregulated in KO myoblasts (Figure 3E). These results underscore irregularities in mitosis, cell cycle arrest and cellular stress in SRSF1‐deficient myoblasts.

### 3.4 SRSF1‐Deficient SCs Exhibit Elevated Rates of Apoptosis During the Perinatal Stage

**FIGURE 4:**
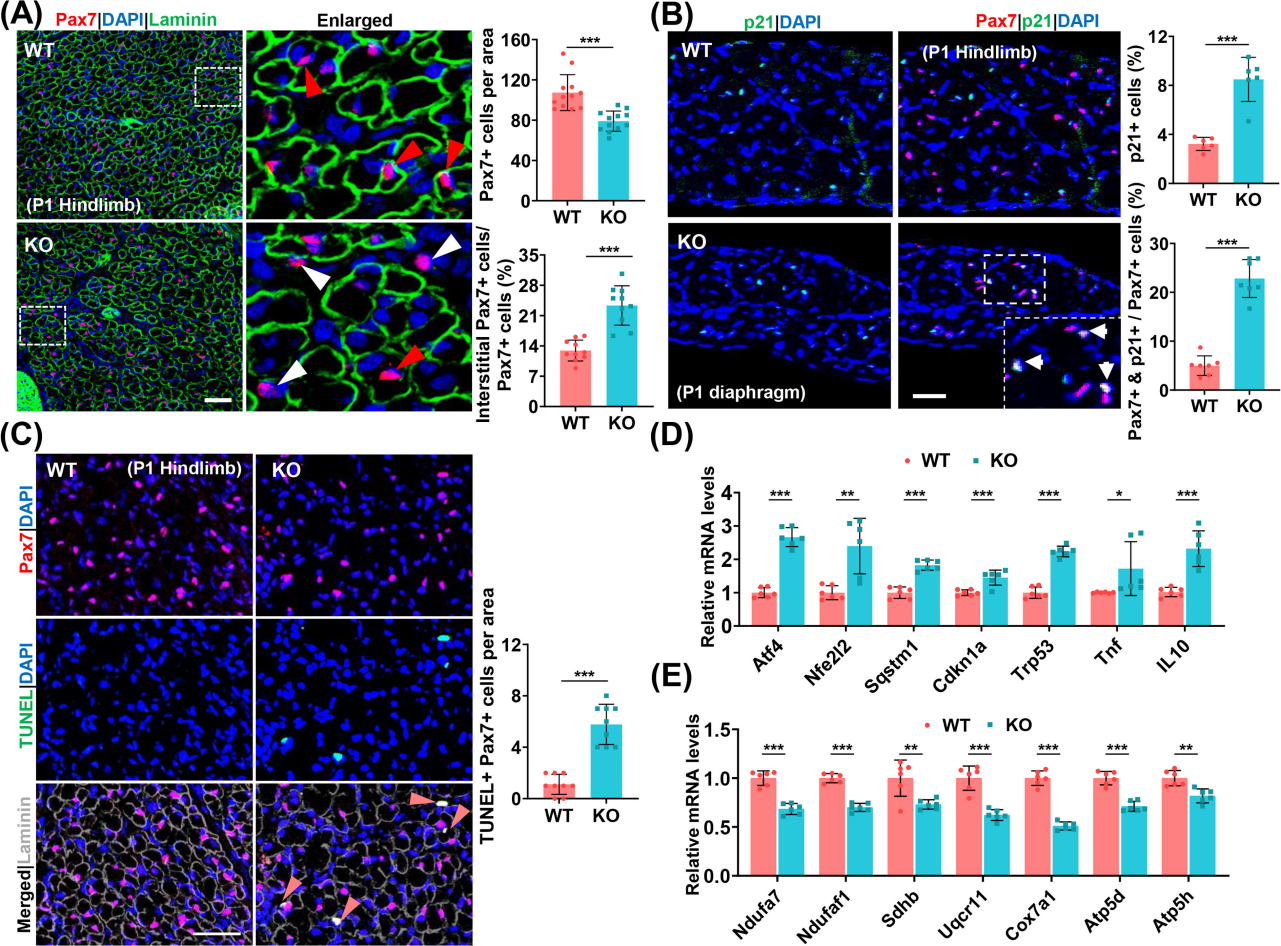
SRSF1‐deficient SCs display improper niche positioning, withdrawal from the cell cycle and elevated apoptosis during the perinatal stage. (A) Hindlimb sections from mice on the first day after birth (P1) were prepared and stained for Pax7 (red), laminin (green) and DAPI (blue). Red arrowheads indicate the satellite cells in the niche location, whereas white arrowheads indicate the interstitial satellite cells. Scale bars, 50 μm. The histograms on the right display the quantification of Pax7 + cells per 0.18 mm^2^ (*n* = 12 per group) and the ratio of interstitial Pax7 + cells to total Pax7 + cells (*n* = 10 per group), respectively. (B) Diaphragm sections from P1 mice were prepared and stained for p21 (green), Pax7 (red) and DAPI (blue) (*n* = 6 per group). The histograms on the right display the quantification of p21 + cells and double Pax7+/p21 + cells, respectively. Scale bars, 25 μm. (C) Immunostaining for TUNEL (green), Pax7 (red), laminin (dark grey) and DAPI (blue) were performed on the hindlimb sections from P1 mice (*n* = 9 per group). Please note that the merging of red, green and blue results in white. Scale bars, 50 μm. The histograms on the right display the quantification of double TUNEL+/Pax7 + cells per 0.18 mm^2^. (D) qRT‐PCR analysis was conducted to examine the expression of stress‐related and inflammation‐related genes in the WT and KO muscles (*n* = 6 per group). (E) qRT‐PCR analysis was conducted to examine the expression of mitochondria‐related genes in the WT and KO muscles (*n* = 6 per group). Results are mean ± SD, *p ≤ 0.05, **p ≤ 0.01, ***p ≤ 0.001 (unpaired Student's *t*‐test).

Mitotic abnormalities and cell cycle arrest are linked to cellular senescence [29, 30, 31]. To this end, we conducted immunostaining on hindlimb sections obtained from newborn mice using Pax7 and laminin antibodies. Both WT mice and heterozygous mice (*Srsf1flox/wt; MyoD‐Cre*) exhibited a similar number of Pax7+ cells (Figure S5). In contrast, KO mice showed a significant reduction in the number of Pax7+ cells compared to controls (Figure 4A). In KO mice, 25% of Pax7+ cells were in the interstitial space, notably higher than the 12% in the control mice, indicating impaired SC homing in the absence of SRSF1. Double immunostaining for p21 and Pax7 in diaphragm sections unveiled increased p21+ cells and Pax7+/p21+ cells in KO mice versus controls (Figure 4B). This suggests that Pax7+ cells in KO mice are irreversibly exiting the cell cycle. Furthermore, TUNEL assays and Pax7 staining on the KO hindlimb sections revealed higher apoptosis rates compared to controls, with the majority of TUNEL+ cells being Pax7+ cells located in the interstitial space (Figure 4C). RT‐qPCR analysis verified increased stress/inflammation‐related gene expression and reduced mitochondrial gene expression in the SRSF1‐deficient muscles (Figure 4D,E). Collectively, these results strongly suggest that loss of SRSF1 induces high rates of apoptosis within SCs during the perinatal stage.

### 3.5 SRSF1 Knockdown Triggers Multipolar Spindle Formation and Cellular Senescence in C2C12 Myoblasts

**FIGURE 5:**
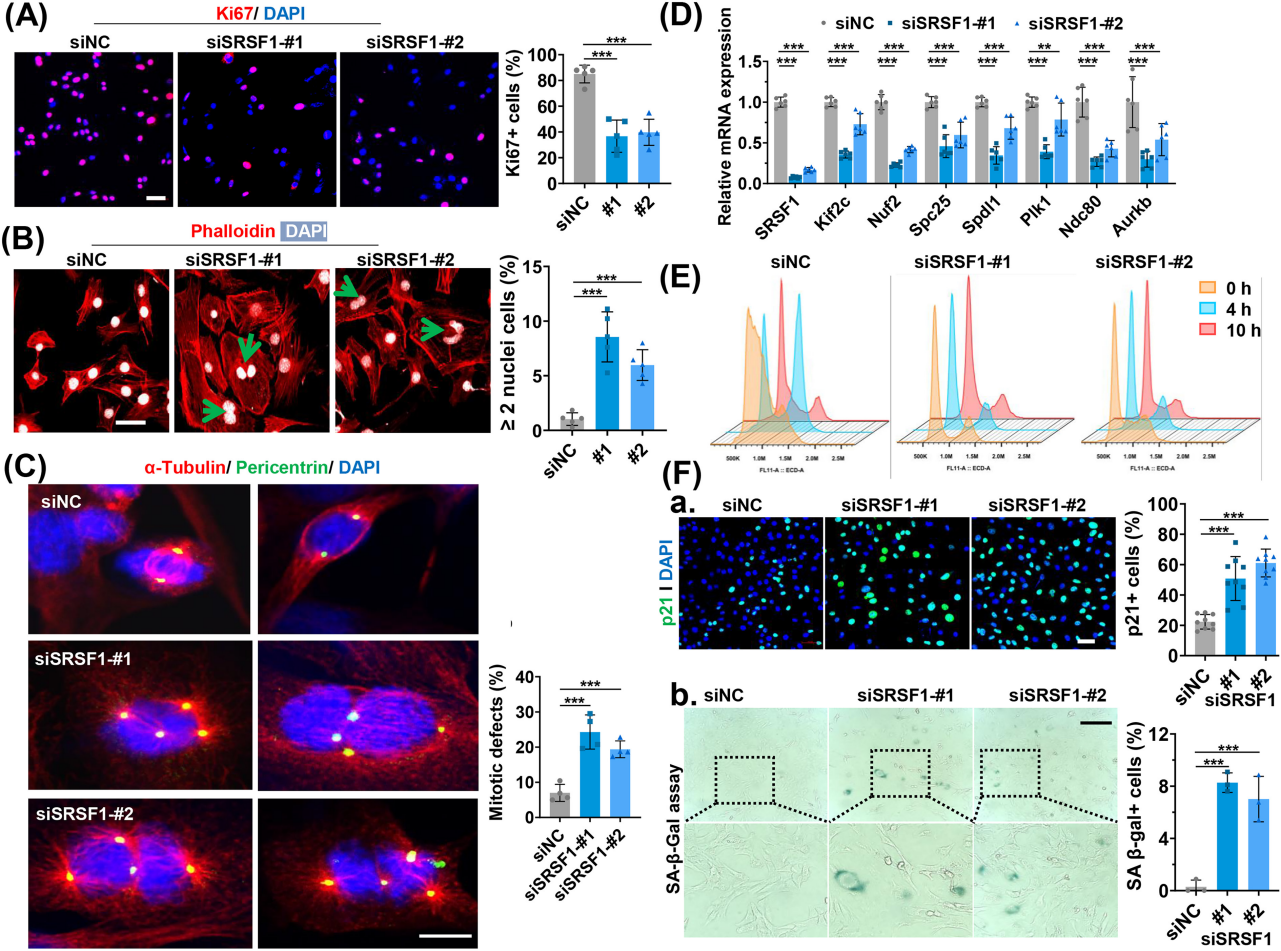
Knockdown of SRSF1 leads to multipolar spindle formation, cell cycle arrest and the onset of cellular senescence in C2C12 myoblasts. (A) Immunostaining for Ki67 (red) and DAPI (blue) was conducted on C2C12 myoblasts after transient transfection with siRNAs against SRSF1 (siSRSF1‐#1 and siSRSF2‐#2) and control siRNA (siNC). Scale bars, 50 μm. The percentage of Ki67+ cells is shown in the right histogram (*n* = 5). (B) Immunostaining of phalloidin (red) and DAPI (white) was performed on C2C12 cells transfected with the indicated siRNA. Binucleated cells are indicated by green arrowheads. Scale bars, 50 μm. The quantification of cells containing more than two nuclei is presented on the right (*n* = 5). (C) Immunostaining of pericentrin (green) and α‐tubulin (red) was carried out on C2C12 cells after transient transfection with the indicated siRNA for 48 h. Scale bars, 10 μm. The quantification of cells with mitotic defects is displayed on the right (*n* = 4). (D) qRT‐PCR analysis was conducted to assess the expression of genes associated with mitotic spindle assembly in C2C12 cells transfected with the indicated siRNA (*n* = 6). (E) Cell cycle distribution was monitored by using flow cytometry. The cells were transfected with the indicated siRNAs and synchronized using a double thymidine block. They were then released into thymidine‐free media for 0 h (orange), 4 h (blue) or 10 h (pink). (F‐a) Immunostaining of p21 (green) and DAPI (blue) was conducted in C2C12 cells after transient transfection for 48 h (top panel). Scale bars, 50 μm. The percentage of p21+ cells is shown on the right (*n* = 9). (F‐b) SA‐β‐gal staining was performed on C2C12 cells (bottom panel). The cells were transfected with the indicated siRNAs, cultured for 5 days and then stained with SA‐β‐gal. Quantification of SA‐β‐gal‐positive cells is displayed on the right (*n* = 3). Scale bars, 200 μm. Results are mean ± SD, *p ≤ 0.05, **p ≤ 0.01, ***p ≤ 0.001 (one‐way ANOVA followed by Dunnett's multiple comparison test).

SRSF1 was highly expressed in growing C2C12 myoblasts and became undetectable after 6 days of differentiation (Figure S6A). Transfections of C2C12 myoblasts with SRSF1‐specific siRNAs (siSRSF1‐#1 and siSRSF1‐#2) showed that SRSF1 knockdown (KD) substantially hampered C2C12 proliferation and differentiation compared to control siRNA (siNC) transfection (Figures 5A and S6B,C). Given the high expression of SRSF1 in proliferative C2C12 myoblasts, we focused its role on proliferation. Importantly, we observed a notable increase in the number of cells with more than two nuclei following treatment with siSRSF1 (Figure 5B). Moreover, our investigation revealed that SRSF1‐KD cells showed a higher occurrence of multipolar spindles and reduced levels of bipolar spindle formation compared to control cells (Figure 5C). This was determined through immunostaining for pericentrin, a marker of centrioles, and α‐tubulin, a marker for microtubules. RT‐qPCR analysis further demonstrated a significant reduction in the expression of genes associated with mitotic spindle assembly in SRSF1‐KD cells compared to control cells (Figure 5D).

The formation of multipolar spindles could lead to cell cycle arrest, cell death or senescence [32]. Therefore, we conducted cell synchronization‐release experiments using a double thymidine block and flow cytometry to analyse cell cycle profiles at different time points after release from block. As shown in Figure 5E, SRSF1 KD resulted in cells being more prone to arrest at G1 or G1/S than control cells at 0 h. Upon release at 4 and 10 h, control cells successfully transitioned from G1/S into G2/M and then back to G1/S phase. However, SRSF1‐KD cells failed to progress from G1/S into G2/M, indicating these cells are irreversible blocked at the G1 phase. In line with cell cycle arrest, p21 levels notably increased in SRSF1‐KD cells (Figure 5F‐a), accompanied by a significant increase in senescent cells following SRSF1 KD, confirmed by SA‐β‐gal staining (Figure 5F‐b). These findings strongly indicate that SRSF1 KD induces the formation of multipolar spindles, leading to cell cycle arrest and triggering cellular senescence in C2C12 myoblasts.

### 3.6 SRSF1‐Mediated Fgfr1op2 Splicing Plays a Crucial Role in Regulating Both Mitotic Spindle Formation and Cell Proliferation

Given SRSF1's known roles as a sequence‐specific splicing activator and a critical AS regulator, we conducted RNA‐seq analysis to identify AS events influenced by SRSF1 in diaphragm muscles. Our results indicated that the genes impacted by SRSF1 were predominantly linked to skeletal muscle physiological function, cell proliferation and protein kinase activity (Figure S7).

**FIGURE 6:**
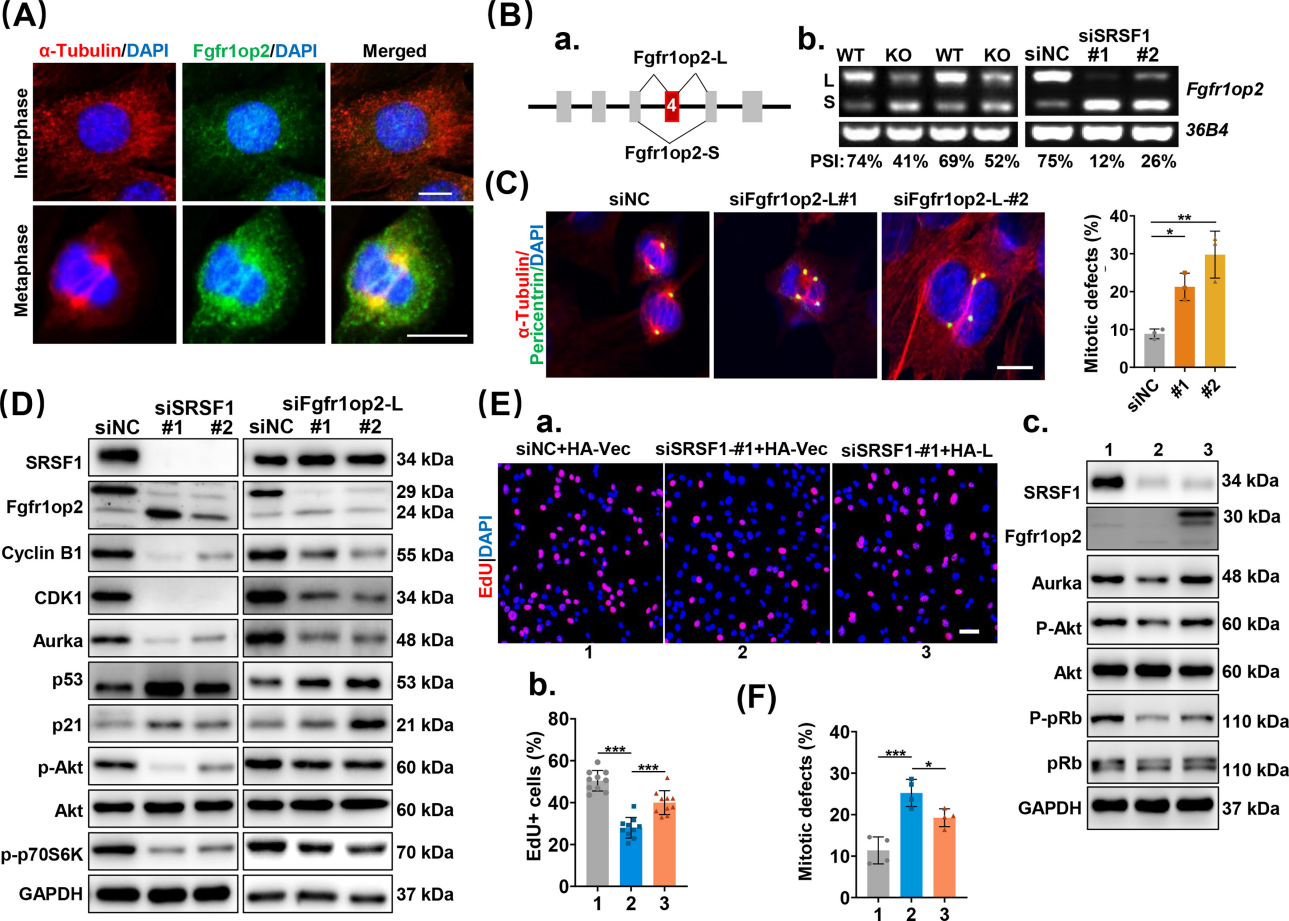
SRSF1‐mediated Fgfr1op2 splicing plays a pivotal role in governing mitotic spindle formation and cell cycle progression. (A) Immunostaining was conducted on C2C12 cells for α‐tubulin (red), Fgfr1op2 (green) and DAPI (blue). The top images depict interphase, whereas the bottom images show metaphase. Scale bars, 10 μm. (B‐a) Schematic diagrams illustrate the two splice variants of Fgfr1op2: the long isoform (L) containing exon 4 and the short isoform (S) lacking exon 4. (B‐b) RT‐PCR results of two Fgfr1op2 isoforms in control and KO diaphragm muscles or control and SRSF1‐KD cells. The percent Spliced In (PSI) values were displayed at the bottom. PSI is calculated as Inclusion/(Inclusion + Exclusion) %. (C) Immunostaining of pericentrin (green) and α‐tubulin (red) was performed on C2C12 cells after transient transfection with siRNAs targeting Fgfr1op2‐L (Fgfr1op2‐#1 and Fgfr1op2‐#2) and siNC. Scale bars, 10 μm. Quantification of cells with mitotic defects is shown on the right (*n* = 3). (D) Whole‐cell lysates were prepared from C2C12 cells transiently transfected with indicated siRNAs and subjected to WB analysis with specified antibodies, along with the molecular weight of each protein in the WB analysis. The protein quantification analyses are presented in the Figure S6A. (E‐a‐b‐c) C2C12 cells were co‐transfected with siRNAs and plasmids, followed by immunostaining and WB analysis. The siRNAs and plasmids used were siNC + pcDNA3.0‐HA‐vector (1), siSRSF1‐#1 + pcDNA3.0‐HA (2) and siSRSF1‐#1 + pcDNA3.0‐HA‐Fgfr1op2‐L. Representative confocal images of EdU (red) and DAPI (blue) staining were presented at the top (a). Scale bars, 50 μm. The histogram below (b) illustrates the percentage of EdU + cells (*n* = 10). Results of WB analysis are depicted in panel (c). The protein quantification analysis of panel (c) is shown in Figure S6B. (F) Immunostaining was conducted on C2C12 cells as described in (E), and quantification of cells with mitotic defects is presented (*n* = 4). Results are mean ± SD; *p ≤ 0.05, **p ≤ 0.01, ***p ≤ 0.001 (one‐way ANOVA for C, E‐b and F, Dunnett's or Tukey's multiple comparison test).

One of the splicing targets that caught our attention is Fgfr1op2, a small molecule associated with cytoskeleton [33]. Immunostaining analysis revealed that although Fgfr1op2 is dispersed through the interphase of C2C12 cells, it primarily localizes to the spindle pole during mitosis, co‐localizing with α‐tubulin, suggesting its potential involvement in mitotic spindle formation (Figure 6A). Fgfr1op2 comprises six exons, with exon 4 undergoing AS regulation, leading to the generation of full‐length (L) and short (S) variants (Figure 6B‐a). The presence of transforming potential in the L‐isoform when fused into Fgfr1 [34] suggests its involvement in proliferation. Interestingly, in diaphragm muscle samples and C2C12 cells, the L‐isoform was predominantly found the WT, whereas the deficiency or KD of SRSF1 resulted in reduced levels of the L‐ isoform (Figure 6B‐b), underscoring the critical role of SRSF1 in regulating the inclusion of Fgfr1op2 exon 4:

To investigate the role of Fgfr1op2 in spindle formation, we designed two independent siRNAs (siL‐1 or siL‐2) targeting exon 4 of Fgfr1op2. Treatment of these siRNAs led to a significant reduction in the levels of the L isoform (Figure S8A). KD of the L isoform resulted in decreased cell proliferation (Figure S8B), increased formation of abnormal mitotic spindles (Figure 6C), reduced expression of spindle assembly‐related genes (Figure S8C) and cell cycle arrest in G1/S phase (Figure S8D). These effects closely resembled those observed in C2C12 cells following SRSF1 KD. Furthermore, both SRSF1 KD and Fgfr1op2‐L KD resulted in reduced protein levels of the functional isoform of Fgfrop2, cell cycle proteins such as Cyclin B1, Cdk1 and Aurka and proliferation‐related proteins p‐Akt and p‐p70S6k, as well as increased levels of p53 and p21 (Figures 6D and S9A).

To delve deeper into the role of the L isoform in mediating the effects of SRSF1, we generated HA‐tagged Fgfr1op2‐L plasmids and conducted a double‐transfection assay in combination with siRNA and HA‐Fgfr1op2‐L plasmids. Cell proliferation was assessed using the EdU incorporation assay. The results demonstrated that the overexpression of Fgfr1op2‐L in SRSF1‐KD cells could restore diminished proliferation compared to the HA‐vector control (Figure 6E‐a‐b). The L isoform restored protein levels observed in SRSF1‐KD cells (Figures 6E‐c and S9B) and reduced the occurrence of multipolar spindles (Figure 6F). These findings highlight the pivotal role of SRSF1‐mediated Fgfr1op2 splicing in regulating mitotic spindle formation and cell proliferation.

### 3.7 The Absence of SRSF1 in Adult QSCs Impedes Their Ability to Proliferate, Leading to a Failure of Muscle Regeneration Following Injury

**FIGURE 7:**
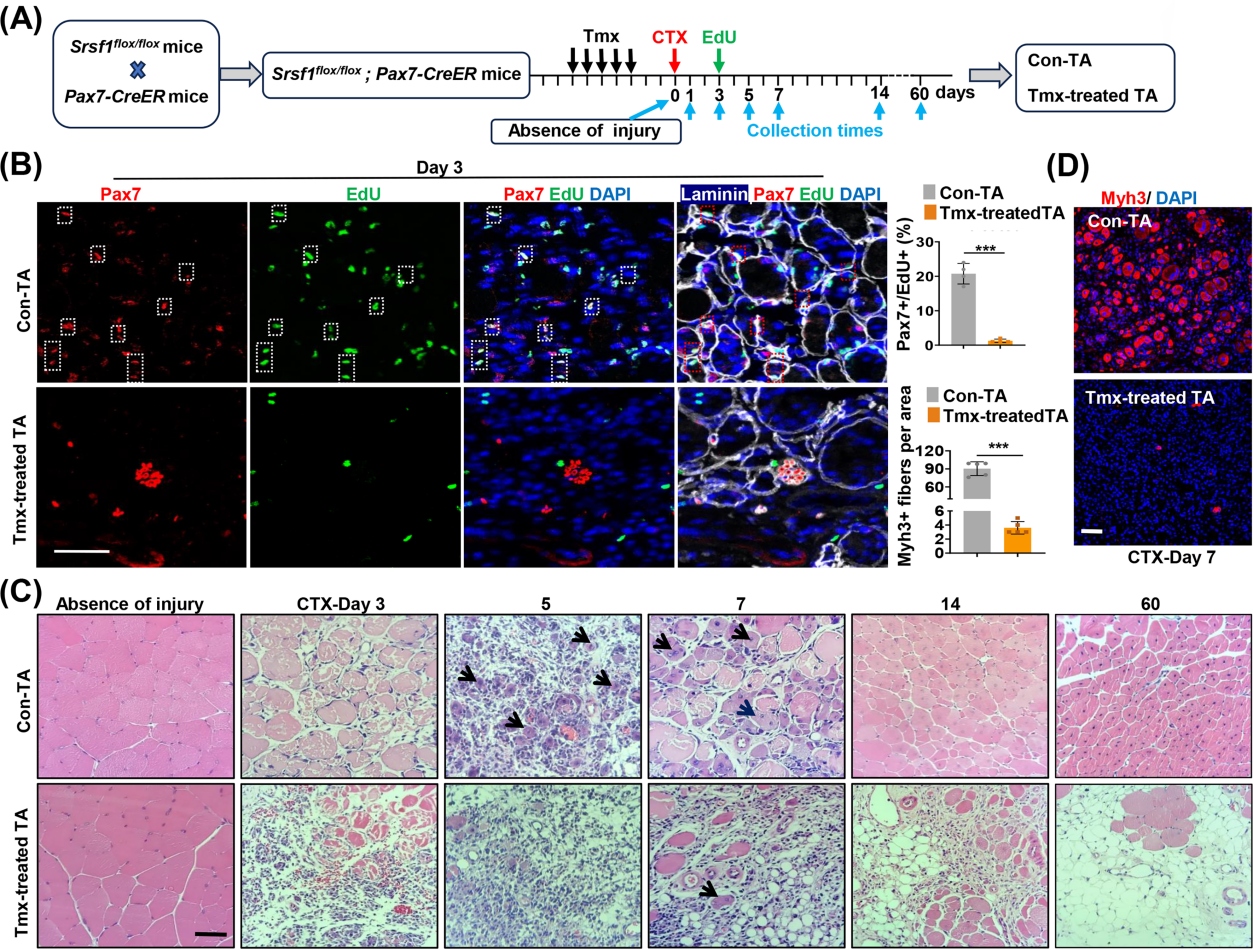
The absence of SRSF1 in adult QSCs impedes their ability to proliferate, leading to a failure of muscle regeneration following injury. (A) Generation of *Srsf1* ^*flox/flox*^ *; Pax7‐CreER* mice, and the experimental strategy involved multiple timelines of interventions, including Tmx injection (as a control with coil oil), CTX injection, EdU injection and collection times of tibialis anterior (TA) muscle samples, both in the absence of injury and after CTX‐induced injury. (B) Representative confocal images of Pax7 (red) and laminin (white) staining in TA muscle sections from control and Tmx‐treated mice on Day 3 after injury. EdU was detected using Alexa‐488 labelling (green) and nuclei were stained with DAPI (blue). Dotted boxes indicated merged EdU+ and Pax7+cells. Scale bars, 50 μm. Quantification of Edu+/Pax7+ cells per area is shown on the right bar graph (*n* = 4 per group). (C) Representative HE images of TA muscles harvested in control and mutant mice at different time points (absence of injury, 3, 5, 7, 14 and 60 days after CTX injury). The new fibres are indicated by the dark arrowheads. Scale bars, 50 μm. (D) Representative confocal images of Myh3 (red) staining with TA muscle sections from control and Tmx‐treated mice on Day 7 after CTX injury. Scale bars, 50 μm. The bar graph in the lower left shows the quantification of Myh3+ myofibres per field (*n* = 5 per group). Results are mean ± SD; *p ≤ 0.05, **p ≤ 0.01, ***p ≤ 0.001 (unpaired Student's *t*‐test).

Given the role of SRSF1 in mitosis progression, we are exploring whether the deletion of SRSF1 impacts adult SC proliferation during skeletal muscle regeneration. To this end, we generated mice with SC‐specific SRSF1 ablation through crossbreeding *Srsf1* ^*flox/flox*^ mice with *Pax7‐CreER* mice. SRSF1 ablation was induced by administering daily intraperitoneal injections of tamoxifen (Tmx) over 5 consecutive days (Figure 7A). We induced muscle injury by administering cardiotoxin (CTX) injections into the TA muscles, followed by the collection and analysis of muscle samples at various time points post‐injury (Figure 7A).

Analysis of Pax7 and MyoD immunostaining data reveals that, in the absence of injury induction, the presence or absence of SRSF1 did not have any discernible effects on the populations of QSCs (Figure S10A) or their spontaneous activation (Figure S10B). Following the initial injury, as observed on Day 1, both groups exhibited limited but comparable levels of MyoD+ cells. However, by Day 5 post‐ injury, a significant increase in the number of MyoD+ cells was evident in the control group. In contrast, the Tmx‐treated group exhibited a notably lower count of MyoD+ cells on Day 5, a count that closely resembled the levels observed on Day 1 (Figure S10B).

The decrease in MyoD+ cell count observed in the Tmx‐treated TA on Day 5 could be attributed to impaired proliferation in the absence of SRSF1. To investigate, both control and Tmx‐treated mice were administered EdU injections on the third day after injury to label proliferating cells for a 3‐h period. As shown in Figure 7B, the control muscles displayed a notable abundance of Pax7+ cells, and the majority of these Pax7+ cells exhibited EdU positivity. In sharp contrast, the Tmx‐treated muscles exhibited a limited number of Pax7+ cells, with almost no cells being EdU+. These findings strongly suggest that the absence of SRSF1 hinders SCs' ability to proliferate effectively in response to injury. Furthermore, histological examination through HE staining revealed prominent differences (Figure 7C). In the control muscles, damaged myofibres were efficiently replaced by newly regenerated fibres with central nuclei on Days 5 and 7 post‐injury (dark arrowheads), and muscle morphology was largely restored by Day 14. Conversely, in the Tmx‐treated muscles, we observed relatively few newly formed fibres, although a significant lipid accumulation was evident on Days 7, 14 and 60. Immunostaining of Myh3 and Desmin further confirmed that the absence of SRSF1 results in a failure of skeletal muscle regeneration (Figures 7D and S10C).

In addition to lipid accumulation following failed regeneration, immunostaining for the macrophage marker CD68 and the T‐cell marker CD3 revealed increased inflammation in Tmx‐treated muscles compared to controls (Figure S11A). However, fibrosis was not observed in either tamoxifen‐treated muscles or controls, revealed by Sirius Red staining (Figure S11B). These results suggested that failed regeneration in the Tmx‐treated muscles was accompanied by increased inflammation and lipid accumulation, but not fibrosis.

Because Tmx is known to affect many aspects of cellular and organisaml physilology [35], to exclude its effects on SRSF1 deletion–mediated muscle regeneration failure in mice, we also treated *Srsf1* ^*flox/flox*^ mice with Tmx and induced muscle damage. Interstingly, these mice displayed similar regeneration phenotypes to *Srsf1* ^*flox/flox*^;*Pax7‐CreER* mice treated with coil oil and subjected to muscle damage (compare Figure S12 with Figure 7), further confirming the critical role of SRSF1 in skeletal muscle regeneration.

### 3.8 SRSF1 Deficiency Induces Multipolar Spindle Formation and Dysregulated Splicing of Fgfr1op2 in Primary Myoblasts

**FIGURE 8:**
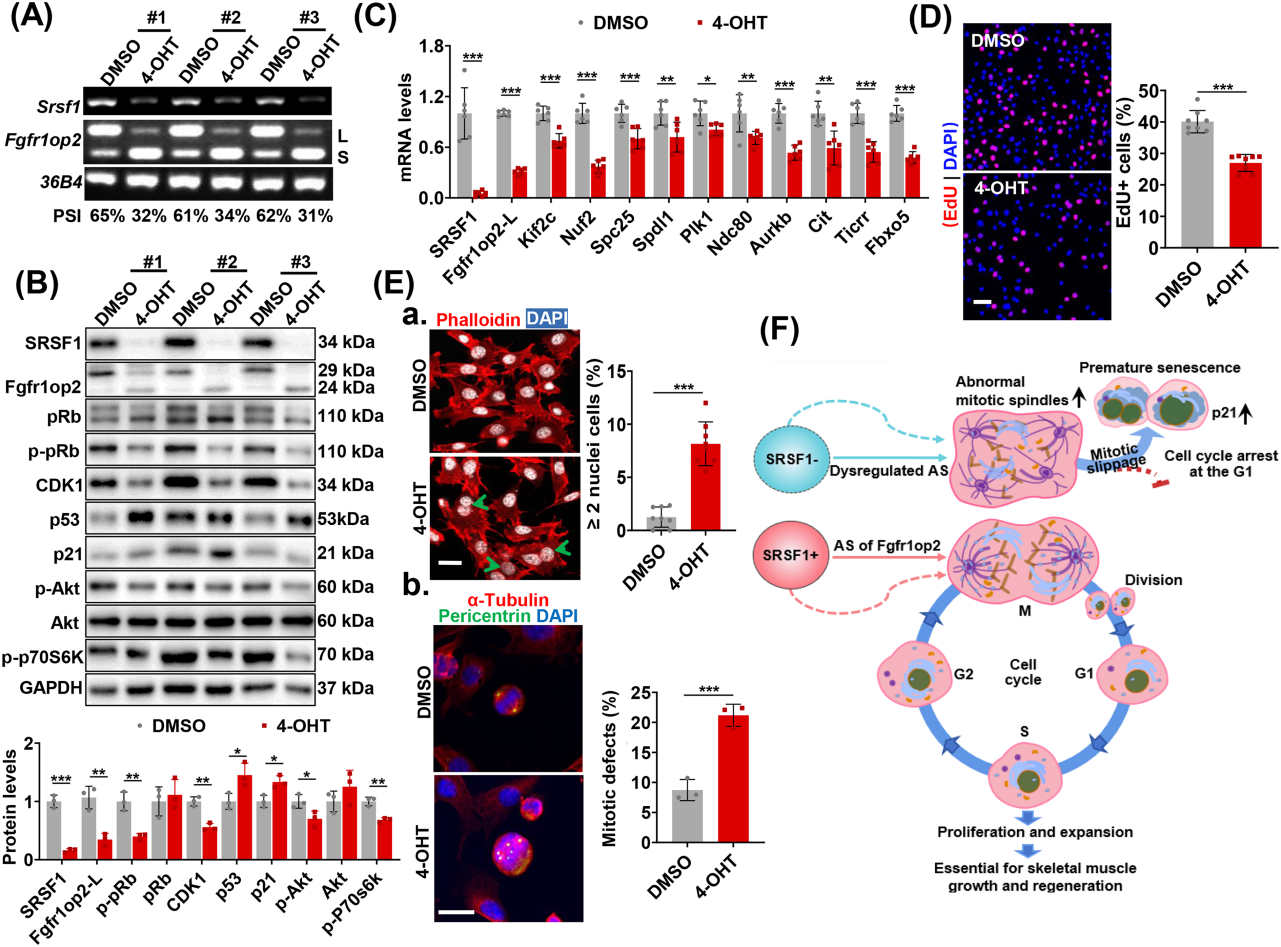
SRSF1 deficiency induces multipolar spindle formation and dysregulated splicing of Fgfr1op2 in primary myoblasts. (A) RT‐PCR showed mRNA levels of SRSF1 and two Fgfr1op2 isoforms in both DMSO‐treated and 4‐OHT‐treated myoblasts. PSI values were presented at the bottom. (B) Whole‐cell lysates from myoblasts treated with DMSO or 4‐OHT were analysed via WB with specific antibodies. The protein quantification analysis is shown at the bottom panel (*n* = 3). (C) qRT‐PCR evaluated gene expression associated with mitotic spindle assembly in DMSO and 4‐OHT‐treated myoblasts (*n* = 6). (D) Immunostaining of EdU (red) and DAPI (blue) in DMSO and 4‐OHT‐treated myoblasts. Scale bar, 50 μm. The percentage of EdU+ cells is presented in the histogram on the right (*n* = 4). (E‐a) Immunostaining of phalloidin (red) and DAPI (white), showing cells with more than two nuclei. Scale bar, 20 μm. The percentage of ≥ 2 nuclei cells is presented in the histogram on the right (*n* = 4). (E‐b) Immunostaining of pericentrin and α‐tubulin in DMSO and 4‐OHT‐treated myoblasts. Scale bar, 20 μm. Quantification of cells with mitotic defects is shown on the right (*n* = 3). (F) A working model illustrates the role of SRSF1‐mediated Fgfr1op2 splicing in regulating satellite cell mitosis, which is essential for satellite cell proliferation and expansion during skeletal muscle growth and regeneration. The absence of SRSF1 caused exon 4 skipping of Fgfr1op2, thereby decreasing the generation of functional proteins, leading to disrupted mitosis, cell cycle arrest and premature senescence. Dashed arrows indicate other splicing targets of SRSF1, as well as additional mechanisms beyond splicing regulation that might be involved in the regulation of mitosis progression in SCs. Results are mean ± SD; *p ≤ 0.05, **p ≤ 0.01, ***p ≤ 0.001 (unpaired Student's *t*‐test).

To investigate the impact of SRSF1 absence on mitosis progression in primary myoblasts, we initiated the process by isolating primary myoblasts from the *Srsf1* ^*flox/flox*^ *; Pax7‐CreER* mice. Subsequently, we subjected these cells to a 4‐hydroxytamoxifen (4‐OHT) treatment for 48 h, which effectively deleted the *Srsf1* gene in the cells. In three independent experiments, both the mRNA and protein levels of SRSF1 exhibited a significant decrease after 4‐OHT treatment compared to the control (Figure 8A,B).

Upon the deletion of SRSF1 from myoblasts, we observed a pronounced reduction in exon4 inclusion of Fgfr1op2, accompanied by a corresponding decrease in the protein isoform (Figure 8A,B). In contrast, neither 4‐OHT nor DMSO treatment affected *Srsf1* mRNA levels or caused exon 4 skipping of Fgfr1op2 in primary myoblasts from *Srsf1* ^*flox/flox*^ mice (Figure S13). Moreover, there were significant reductions in the protein levels of genes implicated in cell cycle regulation and cell proliferation, including Cdk1, p‐Rb, p‐Akt and p‐p70S6k. Conversely, the levels of p53 and p21 exhibited an increase (Figure 8B). Additionally, the expression of genes associated with spindle formation and mitosis progression displayed a notable reduction as well (Figure 8C).

Consistent with the altered gene expression resulting from the deletion of SRSF1 in myoblasts, EdU staining and other immunostaining indicated reduced proliferation, an increase in the number of cells containing more than two nuclei and the presence of abnormal spindles (Figure 8D,E‐a‐b). Collectively, these findings emphasize the central role of SRSF1 and splicing regulation in controlling mitosis in primary myoblasts.

## 4 Discussion

The proliferation of SCs is vital for skeletal muscle growth and regeneration. SRSF1, a splicing factor highly expressed in actively proliferating SCs, plays a pivotal role in this process. Its absence results in significant proliferation defects, observed in both perinatal and adult SCs upon injury in mice. SRSF1 controls mitosis progression and prevents premature senescence in SCs through its AS regulation, particularly regarding Fgfr1op2 (Figure 8F).

Extensive scientific literature explores the mechanisms responsible for maintaining the quiescent state of SCs. These studies have identified numerous quiescence‐maintaining factors, such as Pten [36], Notch [37, 38] and Spry1 [14]. These factors are highly enriched and functionally active in QSCs, where they assume a pivotal role in safeguarding their dormant state. Deletion of these factors results in SC depletion due to spontaneous differentiation, impaired SC proliferation and motility, premature cell cycle entry or apoptosis.

SRSF1, in contrast to these factors, is notably absent in QSCs but highly expressed in proliferating myoblasts [23]. Our scRNA‐seq analysis has unveiled significant heterogeneity within the perinatal SC population. These findings parallel the observed heterogeneity within human induced pluripotent stem cell–derived muscle progenitors [39]. The absence of SRSF1 within MyoD+ progenitors resulted in mitosis abnormalities, ultimately causing cell cycle arrest at the G1 phase. These observations shed light on the increased prevalence of activated and proliferating myoblasts within the SC population when SRSF1 is lacking. Consistently, SRSF1‐deficient SCs were observed in an improper niche position and undergoing cell cycle withdrawal. Taken together, these findings underscore the critical role of SRSF1 in governing mitosis progression and proliferation of SCs.

The role of SRSF1 in mitosis progression within perinatal SCs was substantiated through KD experiments conducted in C2C12 myoblasts. The KD of SRSF1 resulted in the formation of multipolar spindles, G1‐phase cell cycle arrest and the induction of cellular senescence. These observations are consistent with the phenomenon known as mitotic catastrophe, as described by Vitale et al. [32] Moreover, in vitro culturing of primary myoblasts provided additional evidence of mitotic abnormalities following SRSF1 deletion. This provides an explanation for the observed lack of proliferation in adult QSCs following injury in the absence of SRSF1, ultimately leading to a complete failure of muscle regeneration.

A reduction in p‐Akt and p‐p70S6K levels was observed in both C2C12 and primary myoblasts following SRSF1 deletion, both of which play crucial roles in cell proliferation. Notably, Igf1 is known to activate the PI3K/Akt pathway, leading to the phosphorylation and activation of Akt1 in SCs [40]. Hence, the reduced levels of p‐Akt and p‐p70S6k in myoblasts mirror the decreased expression of Igf1 in SCs lacking SRSF1, as revealed by scRNA‐seq (Figure S14). However, there was no significant changes observed in the MAPK and Fgf pathways upon SRSF1 KD (Figure S15), despite scRNA‐seq showing an upregulation. This suggests the possibility of a feedback response in the context of SRSF1 deficiency.

Given its established role as a critical regulator of AS events [18, 19, 20], it is not surprising that SRSF1 primarily functions by modulating AS regulation, particularly in Fgfr1op2, within muscle stem cells. SRSF1 promotes exon inclusion, generating the full‐length functional protein that predominates in myoblasts and skeletal muscles. Indeed, the co‐localization of Fgfr1op2 with α‐tubulin at the spindle pole during metaphase and the observed multipolar spindles upon Fgfr1op2 KD provide strong evidence for its critical role in mitotic spindle formation. Importantly, its overexpression successfully rescued proliferation defects induced by SRSF1 KD. These results emphasize the vital role of the functional isoform of Fgfr1op2 and SRSF1‐mediated splicing of Fgfr1op2 in mitotic spindle formation and the regulation of cell proliferation.

In summary, our findings demonstrated that SRSF1 is vital for preserving the functionality of SCs and maintaining the regenerative potential of skeletal muscle. These findings hold significant implications for the development of therapeutic strategies for muscular dystrophy and for the prevention of muscle decline.

## Conflicts of Interest

The authors declare no conflicts of interest.

## Supporting information


**Figure S1** Violin plots showing the percentage of mitochondrial counts per cell (percent.mito), the number of genes per cell (nGene) and the UMIs per cell (nUMI) between WT and KO samples after quality control.
**Figure S2.** (A)Heatmap showing the expression of top 10 cell‐type specific markers among six SKM subclusters. (B)Violin plots displaying the expression levels of Myot and Tmod4 among six SKM subclusters. (C) RNA velocity analysis of six SKM subclusters.
**Figure S3.** Heatmaps showing the top 20 differential expression of genes between sC3 and sC1, as well as sC3 and sC2 are presented, and summary table below details the number of down‐regulated genes and upregulated genes in sC3 relative to sC1 or sC2 in below table.
**Figure S4.** Inactivation of SRSF1 led to impaired differentiation. Violin plots demonstrated the expression levels of indicated genes in sC4 between WT and KO groups.
**Figure S5.** Comparable numbers of Pax7+ cells were observed in P1 WT and Het (Srsf1 flox/wt; MyoDCre) mice. Hindlimb sections from mice on the first day after birth (P1) were prepared and stained for Pax7 (red), Laminin (green), and DAPI (blue). Scale bars, 50 μm. The histograms on the right display the quantification of Pax7+ cells per area (*n* = 5 per group).
**Figure S6.** SRSF1 was highly expressed in growing C2C12 myoblasts and essential for cell differentiation. (A). C2C12 myoblasts were cultured in growing medium (GM) and switched to differentiation medium (DM) for differentiation. Whole cell lysates were isolated and subjected to WB analysis. (B, C) Immunostaining for Myogenin (green) or MHC (red) was conducted on C2C12 myoblasts after 3 days and 5 days of differentiation. Cells were transiently transfected with siRNAs against SRSF1 for 48 h, and then induced into differentiation for 3 days (for B) or 5 days (for C). Scale bars, 100 μm. The bar graphs on the right shows the quantification of Myogenin+ cells or Myotubes per field (*n* = 3 per group). Results are Mean ± SD, *p ≤ 0.05, **p ≤ 0.01, ***p ≤ 0.001 (One‐way ANOVA for B, C, Bonferroni’s multiple comparison test).
**Figure S7.** Representative AS events regulated by SRSF1 in the P1 diaphragm muscle (A‐C). Validation of representative exon inclusion or exclusion events influenced by SRSF1 was conducted through RT‐PCR on the three pairs of samples obtained from WT and KO diaphragms. The inclusion and skipping of alternative exons were indicated on the right as E + or E‐, respectively, along with the exon number in each gene. The Precent Spliced In (PSI) values were presented at the bottom.
**Figure S8.** The knockdown of the L isoform resulted in decreased cell proliferation. (A) RT‐PCR results showed a significant decrease in Fgfr1op2‐L after transfected with siRNAs against Fgfr1op2‐L (Fgfr1op2‐#1 and Fgfr1op2‐#2) and siNC. (B) Immunostaining of Ki67 (red) and DAPI (blue) in C2C12 cells after transiently transfected with siRNAs shown. Scale bars, 50 μm. The percentage of Ki67+ cells was shown on the right histogram (*n* = 6). (C) qRT‐PCR analysis examined the expression of genes associated with mitotic spindle organization in C2C12 cells transfected with the indicated siRNA (n = 6). (D) Cell cycle analysis was conducted in C2C12 cells transiently transfected with indicated siRNAs and synchronized using a double thymidine block. The cells were then released into thymidine‐free media for 0 h (orange), 4 h (blue) or 10 h (pink). Results are Mean ± SD, *p ≤ 0.05, **p ≤ 0.01, ***p ≤ 0.001 (one‐way ANOVA followed by Dunnett’s multiple comparison test).
**Figure S9.** The protein quantification analysis from Figure 6. (A) The protein quantification analysis from Figure 6 (D) was conducted using imageJ (*n* = 3). (B) The protein quantification analysis from Figure 6 (E) c was conducted using imageJ (n = 3). Results are Mean ± SD, *p ≤ 0.05, **p ≤ 0.01, ***p ≤ 0.001 (one‐way ANOVA, Dunnett’s multiple comparison test).
**Figure S10.** Absence of SRSF1 in adult SCs results in muscle regeneration failure. (A) Immunostaining of Pax7 (red) and Laminin (white) in TA muscle sections from control and Tmx‐treated mice in the absence of injury. Scale bars, 25 μm. The bar graph on the right shows the quantification of Pax7+ cells per field (0.18 mm2) (*n* = 4 per group). (B) Representative confocal images of MyoD (red) staining in TA muscle sections from control and Tmx‐treated mice in the absence of injury, on day 1 and on day 5 after injury. Scale bars, 50 μm. The bar graph on the right shows the quantification of MyoD+ cells per field between the two groups on day1 and day 5 after injury (*n* = 4 per group). (C) Immunostaining of Laminin (green), Desmin (red) and DAPI (blue) with TA muscle sections from control and Tmx‐treated mice on day 7 after CTX injury. Scale bars, 50 μm. The bottom bar graph below the images shows the quantification of Desmin+ cells per field (*n* = 5 per group). Results are Mean ± SD, *p ≤ 0.05, **p ≤ 0.01, ***p ≤ 0.001 (Student’s t‐test for A and C, and two‐way ANOVA for B, Bonferroni’s multiple comparison test).
**Figure S11.** Increased inflammatory response was observed in Tmx‐treated TA muscles. (A) Immunostaining of CD68 and CD3 in TA muscle sections from control and Tmx‐treated mice on day3 and 7 after injury. Scale bars, 50 μm. The bar graphs on the right shows the quantification of CD68 + cells or CD3 + cells per field (0.18 mm2) (*n* = 5 per group for CD68 staining and *n* = 3 for CD3 staining). (B) Representative images of Sirius red staining in TA muscle sections from control and Tmx‐treated mice on day 7 after injury. Scale bars, 250 μm. Results are Mean ± SD, *p ≤ 0.05, **p ≤ 0.01, ***p ≤ 0.001 (Two‐way ANOVA for A, Bonferroni’s multiple comparison test).
**Figure S12.** Srsf1 flox/flox mice treated with tamoxifen successfully resored muscle regeneration following mucle injury. (A) The experimental strategy involved multiple timelines of interventions, including Tmx injection, CTX injection, EdU injection, and collection times of TA samples, both in the absence of injury and after CTX‐induced injury in Srsf1 flox/flox mice. (B) Immunostaining of Pax7 (red) and Laminin (white) staining in TA muscle sections on day 3 after injury. EdU was detected using Alexa‐488 labeling (green) and nuclei were stained with DAPI (blue). Dotted boxes indicated merged EdU+ and Pax7+cells. Scale bars, 50 μm. Quantification of Edu+/Pax7+ cells per area is shown on the right bar graph (*n* = 4 per group). (C) Immunostaining of Laminin (green), Desmin (red) and DAPI (blue) in TA muscle sections on day 7 after CTX injury. Scale bars, 50 μm. The right bar graph shows the quantification of Desmin+ myofibers per field (*n* = 5 per group). (D) Immunostaining of Myh3 (red) in TA muscle sections on day 7 after CTX injury. Scale bars, 50 μm. The right bar graph shows the quantification of Myh3+ myofibers per field (*n* = 4 per group). (E) Representative HE images of TA muscles harvested at different time points (absence of injury, 3, 7, 14, and 21 days after CTX injury. Scale bars, 50 μm. Results are Mean ± SD.
**Figure S13.** 4‐OHT treatment doesn’t cause exon 4 skipping of Fgfr1op2 in primary myoblasts from Srsf1 flox/flox mice. RT‐PCR showed mRNA levels of Srsf1 and two Fgfr1op2 isoforms in both DMSO‐treated and 4‐OHT‐treated myoblasts. PSI values were presented at the bottom.
**Figure S14.** Reduced Igf1 levels in subclusters of KO group. Violin plots demonstrated the expression levels of Igf1 in subclusters between WT and KO groups.
**Figure S15.** No changes were observed in the Fgf and MAPK pathway following the knockdown of SRSF1 or the Fgfr1op2‐L isoform. C2C12 myoblasts were transiently transfected with the indicated siRNA for 48 h. basic FGF (PeproTech, 100‐18B) was added for 2 min, and cells were harvested for WB analysis using the indicated antibodies.
**Table S1.** Primer sequences used for mice genotyping
**Table S2.** Antibodies used in WB and IF
**Table S3.** Primer sequences used for mRNA expression analysis
**Table S4.** siRNA sequences used for RNA interference
