# Supplementary material for: SRSF1 Is Crucial for Maintaining Satellite Cell Homeostasis During Skeletal Muscle Growth and Regeneration
Source: J Cachexia Sarcopenia Muscle. 2024 Oct 9;15(6):2629–41. doi: 10.1002/jcsm.13607 (PMC11634495; doi:10.1002/jcsm.13607)
Supplement: Supplementary file 1 — Figure S1 Violin plots showing the percentage of mitochondrial counts per cell (percent.mito), the number of genes per cell (nGene) and the UMIs per cell (nUMI) between WT and KO samples after quality control. Figure S2. (A)Heatmap showing the expression of top 10 cell‐type specific markers among six SKM subclusters. (B)Violin plots displaying the expression levels of Myot and Tmod4 among six SKM subclusters. (C) RNA velocity analysis of six SKM subclusters. Figure S3. Heatmaps showing the top 20 differential expression of genes between sC3 and sC1, as well as sC3 and sC2 are presented, and summary table below details the number of down‐regulated genes and upregulated genes in sC3 relative to sC1 or sC2 in below table. Figure S4. Inactivation of SRSF1 led to impaired differentiation. Violin plots demonstrated the expression levels of indicated genes in sC4 between WT and KO groups. Figure S5. Comparable numbers of Pax7+ cells were observed in P1 WT and Het (Srsf1 flox/wt; MyoDCre) mice. Hindlimb sections from mice on the first day after birth (P1) were prepared and stained for Pax7 (red), Laminin (green), and DAPI (blue). Scale bars, 50 μm. The histograms on the right display the quantification of Pax7+ cells per area (n = 5 per group). Figure S6. SRSF1 was highly expressed in growing C2C12 myoblasts and essential for cell differentiation. (A). C2C12 myoblasts were cultured in growing medium (GM) and switched to differentiation medium (DM) for differentiation. Whole cell lysates were isolated and subjected to WB analysis. (B, C) Immunostaining for Myogenin (green) or MHC (red) was conducted on C2C12 myoblasts after 3 days and 5 days of differentiation. Cells were transiently transfected with siRNAs against SRSF1 for 48 h, and then induced into differentiation for 3 days (for B) or 5 days (for C). Scale bars, 100 μm. The bar graphs on the right shows the quantification of Myogenin+ cells or Myotubes per field (n = 3 per group). Results are Mean ± SD, *p ≤ 0.05, **p ≤ [file JCSM-15-2629-s001.pdf]

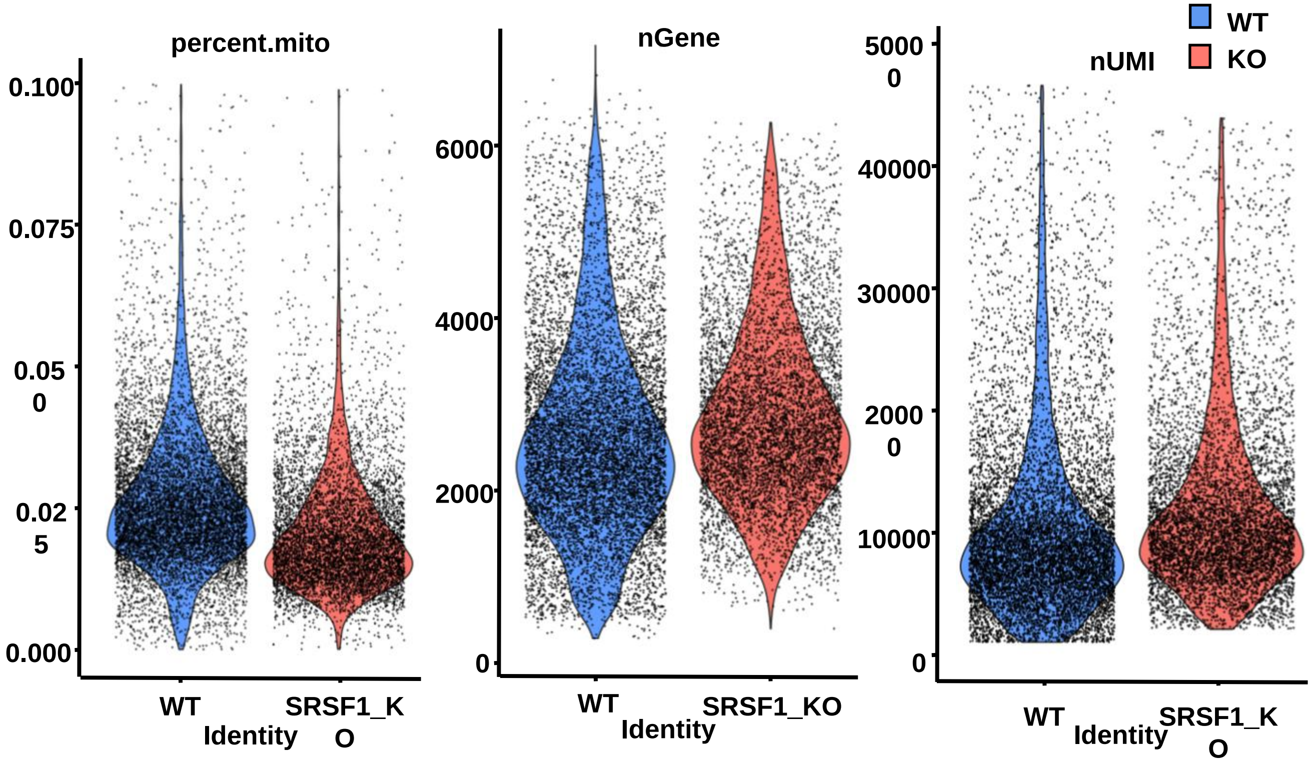

Figure S1. Violin plots showing the percentage of mitochondrial counts per cell (percent.mito), the number of genes per cell (nGene) and the UMIs per cell (nUMI) between WT and KO samples after quality control.

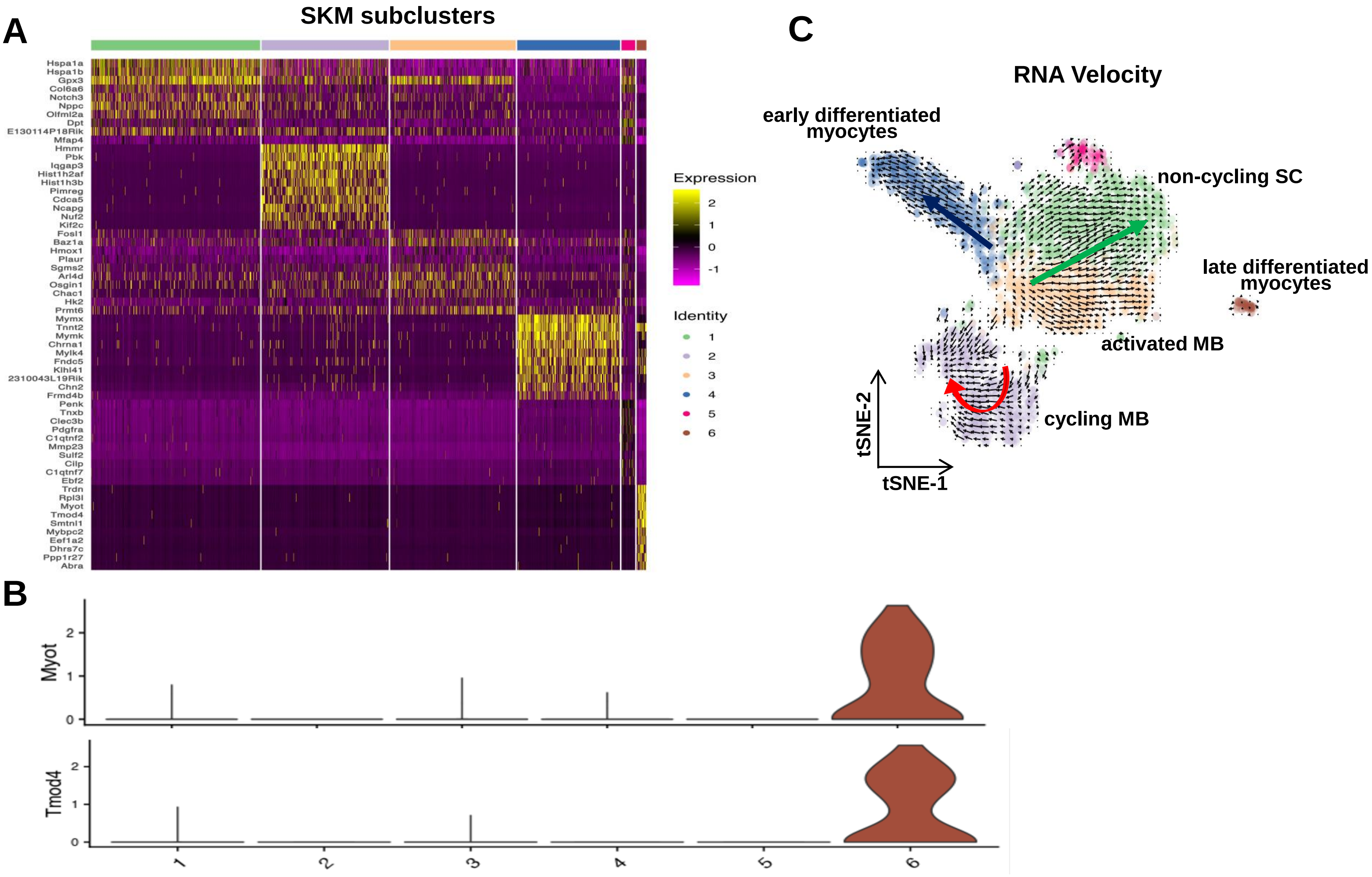

Figure S2. (A) Heatmap showing the expression of top 10 cell-type specific markers among six SKM subclusters. (B) Violin plots displaying the expression levels of *Myot* and *Tmod4* among six SKM subclusters. (C) RNA velocity analysis of six SKM subclusters.

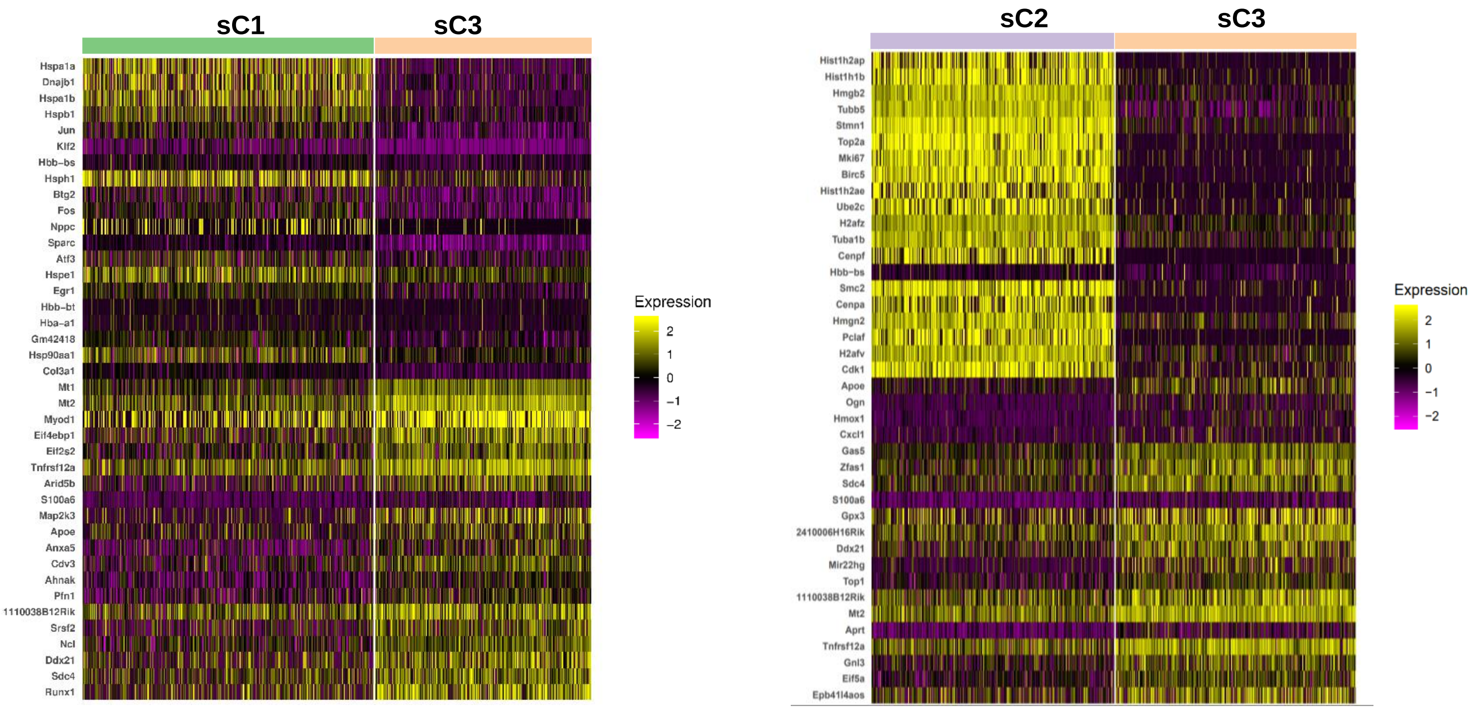

	Total diff-gene (pvalue<0.05, Foldchange>1.5)	
sC3 vs sC1	79	Up: 45
		Down: 34
sC3 vs sC2	197	Up: 21
		Down: 176

Figure S3. Heatmaps showing the top 20 differential expression of genes between sC3 and sC1, as well as sC3 and sC2 are presented, and summary table below details the number of down-regulated genes and up-regulated genes in sC3 relative to sC1 or sC2 in below table.

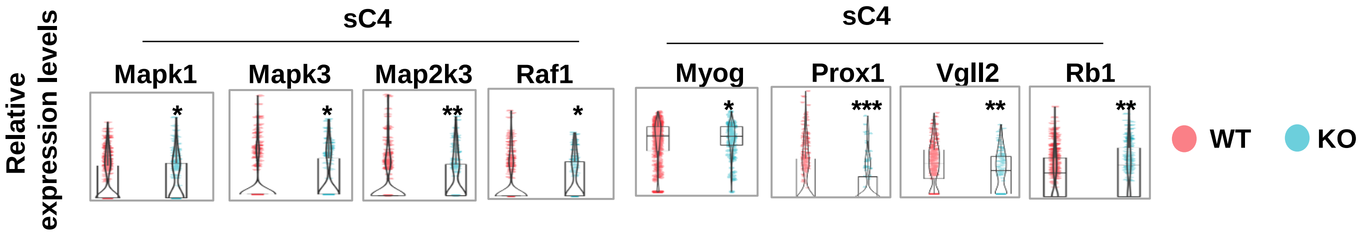

Figure S4. Inactivation of SRSF1 led to impaired differentiation. Violin plots demonstrated the expression levels of indicated genes in sC4 between WT and KO groups.

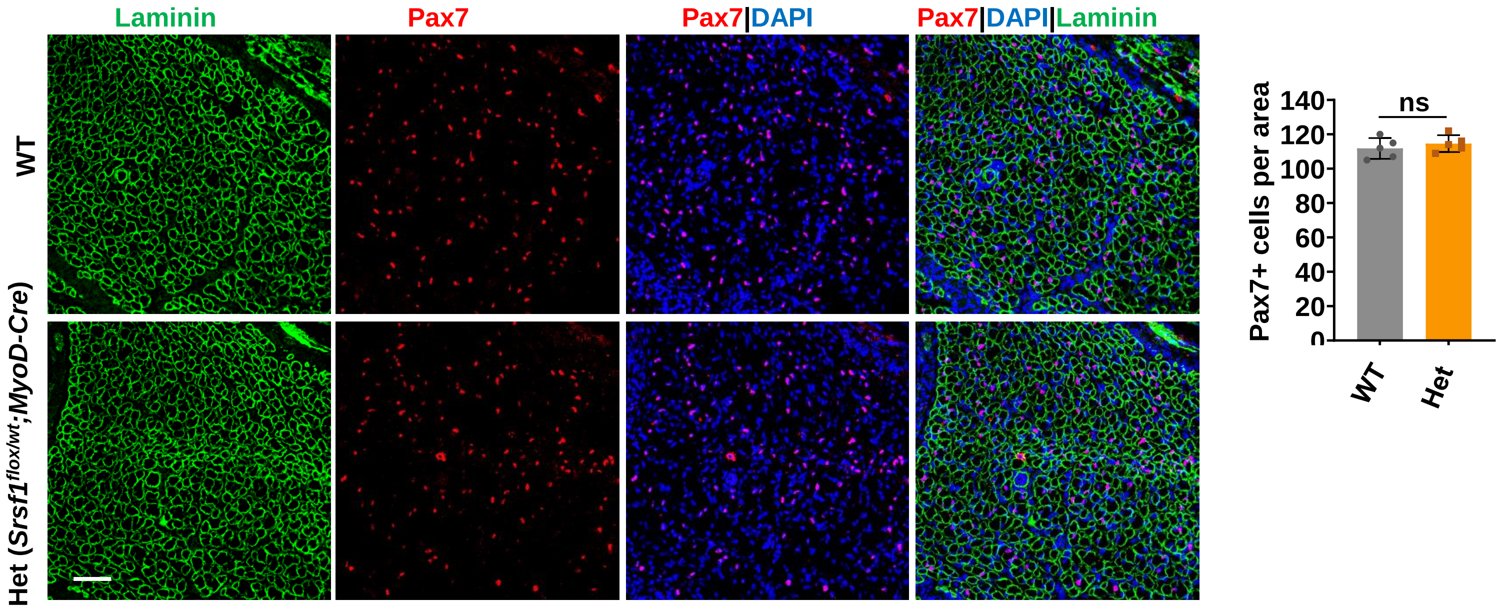

Figure S5. Comparable numbers of Pax7+ cells were observed in P1 WT and Het *(Srsf1$^{flox/wt}$; MyoD-Cre)* mice. Hindlimb sections from mice on the first day after birth (P1) were prepared and stained for Pax7 (red), Laminin (green), and DAPI (blue). Scale bars, 50 µm. The histograms on the right display the quantification of Pax7+ cells per area (n=5 per group).

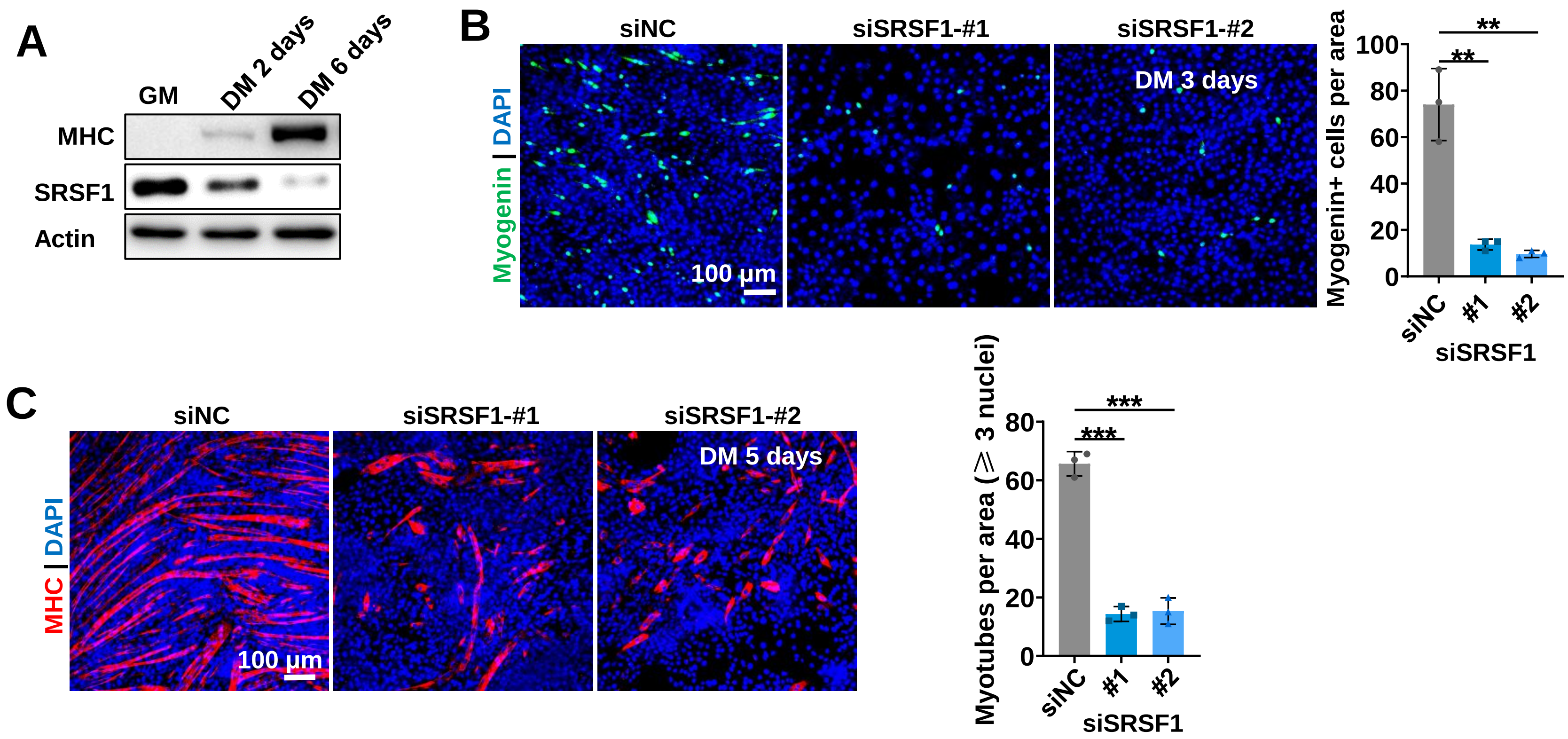

Figure S6. SRSF1 was highly expressed in growing C2C12 myoblasts and essential for cell differentiation. (A). C2C12 myoblasts were cultured in growing medium (GM) and switched to differentiation medium (DM) for differentiation. Whole cell lysates were isolated and subjected to WB analysis. (B, C) Immunostaining for Myogenin (green) or MHC (red) was conducted on C2C12 myoblasts after 3 days and 5 days of differentiation. Cells were transiently transfected with siRNAs against SRSF1 for 48h, and then induced into differentiation for 3 days (for B) or 5 days (for C). Scale bars, 100 μm. The bar graphs on the right shows the quantification of Myogenin+ cells or Myotubes per field (n=3 per group). Results are Mean ± SD, *P ≤ 0.05, **P ≤ 0.01, ***P ≤ 0.001 (One-way ANOVA for B, C, Bonferroni's multiple comparison test).

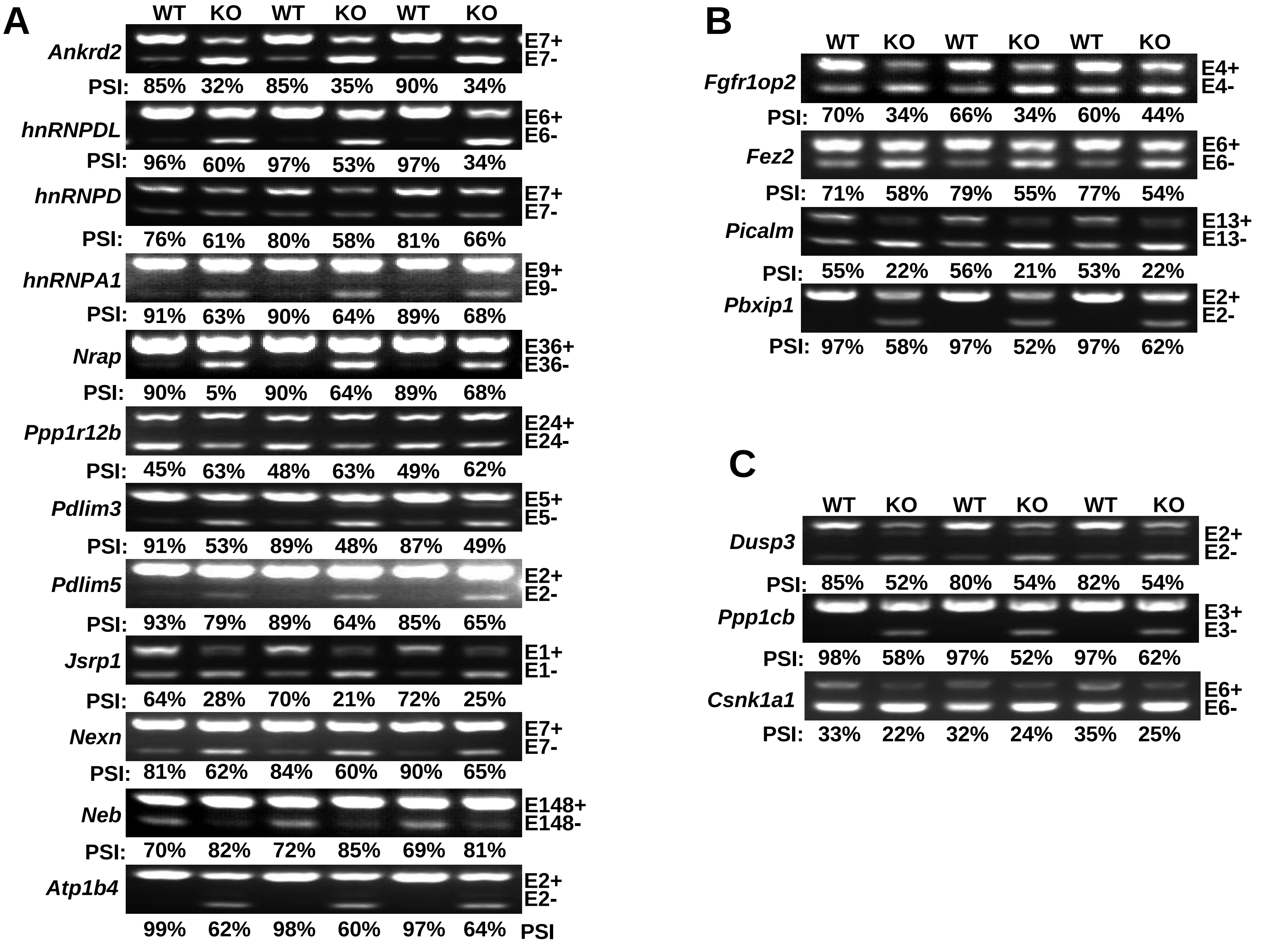

Figure S7. Representative AS events regulated by SRSF1 in the P1 diaphragm muscle (A-C). Validation of representative exon inclusion or exclusion events influenced by SRSF1 was conducted through RT-PCR on the three pairs of samples obtained from WT and KO diaphragms. The inclusion and skipping of alternative exons were indicated on the right as E+ or E- , respectively, along with the exon number in each gene. The Precent Spliced In (PSI) values were presented at the bottom.

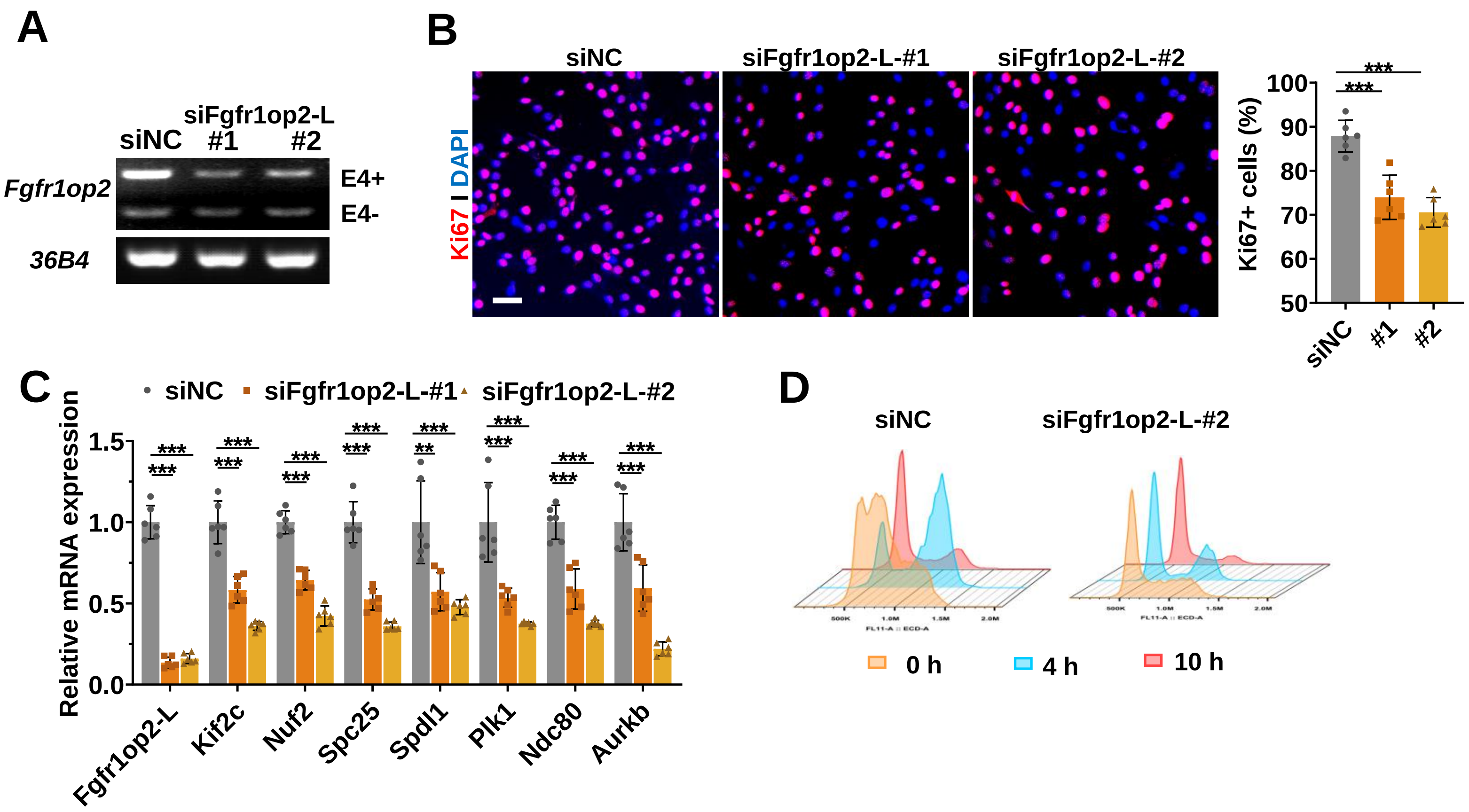

Figure S8. The knockdown of the L isoform resulted in decreased cell proliferation. (A) RT-PCR results showed a significant decrease in Fgfr1op2-L after transfected with siRNAs against Fgfr1op2-L (Fgfr1op2-#1 and Fgfr1op2-#2) and siNC. (B) Immunostaining of Ki67 (red) and DAPI (blue) in C2C12 cells after transiently transfected with siRNAs shown. Scale bars, 50 μm. The percentage of Ki67+ cells was shown on the right histogram (n=6). (C) qRT-PCR analysis examined the expression of genes associated with mitotic spindle organization in C2C12 cells transfected with the indicated siRNA (n=6). (D) Cell cycle analysis was conducted in C2C12 cells transiently transfected with indicated siRNAs and synchronized using a double thymidine block. The cells were then released into thymidine-free media for 0 hour (orange), 4 hour (blue) or 10 hour (pink). Results are Mean ± SD, *P ≤ 0.05, **P ≤ 0.01, ***P ≤ 0.001 (one-way ANOVA followed by Dunnett’s multiple comparison test).

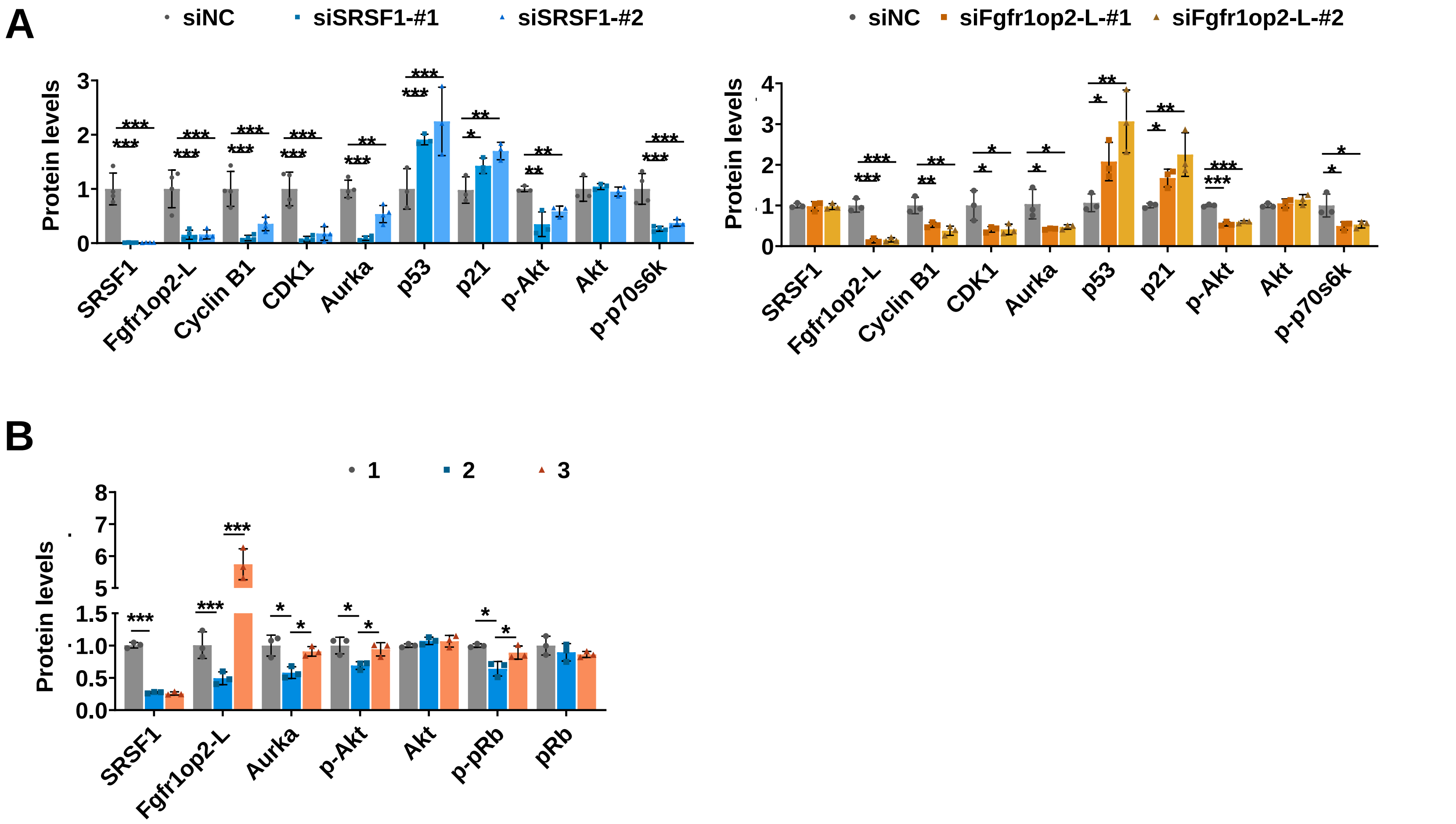

Figure S9. The protein quantification analysis from Figure 6. (A) The protein quantification analysis from Figure 6 (D) was conducted using imageJ (n=3). (B) The protein quantification analysis from Figure 6 (E) c was conducted using imageJ (n=3). Results are Mean ± SD, *P ≤ 0.05, **P ≤ 0.01, ***P ≤ 0.001 (one-way ANOVA, Dunnett's multiple comparison test).

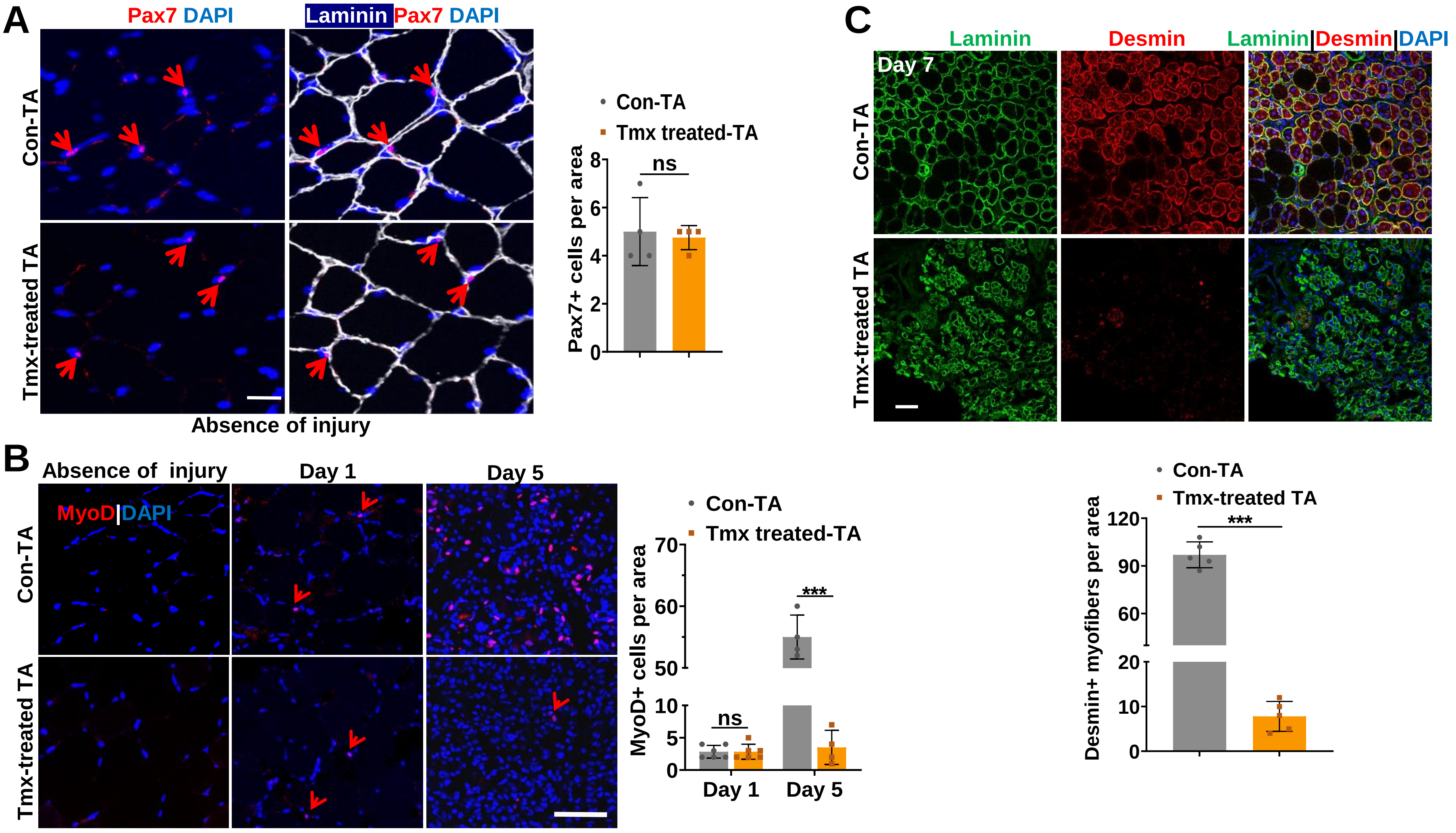

Figure S10. Absence of SRSF1 in adult SCs results in muscle regeneration failure. (A) Immunostaining of Pax7 (red) and Laminin (white) in TA muscle sections from control and Tmx-treated mice in the absence of injury. Scale bars, 25 μm. The bar graph on the right shows the quantification of Pax7+ cells per field (0.18 mm^2) (n=4 per group). (B) Representative confocal images of MyoD (red) staining in TA muscle sections from control and Tmx-treated mice in the absence of injury, on day 1 and on day 5 after injury. Scale bars, 50 μm. The bar graph on the right shows the quantification of MyoD+ cells per field between the two groups on day1 and day 5 after injury (n=4 per group). (C) Immunostaining of Laminin (green), Desmin (red) and DAPI (blue) with TA muscle sections from control and Tmx-treated mice on day 7 after CTX injury. Scale bars, 50 μm. The bottom bar graph below the images shows the quantification of Desmin+ cells per field (n=5 per group). Results are Mean ± SD, *P ≤ 0.05, **P ≤ 0.01, ***P ≤ 0.001 (Student's *t*-test for A and C, and two-way ANOVA for B, Bonferroni's multiple comparison test).

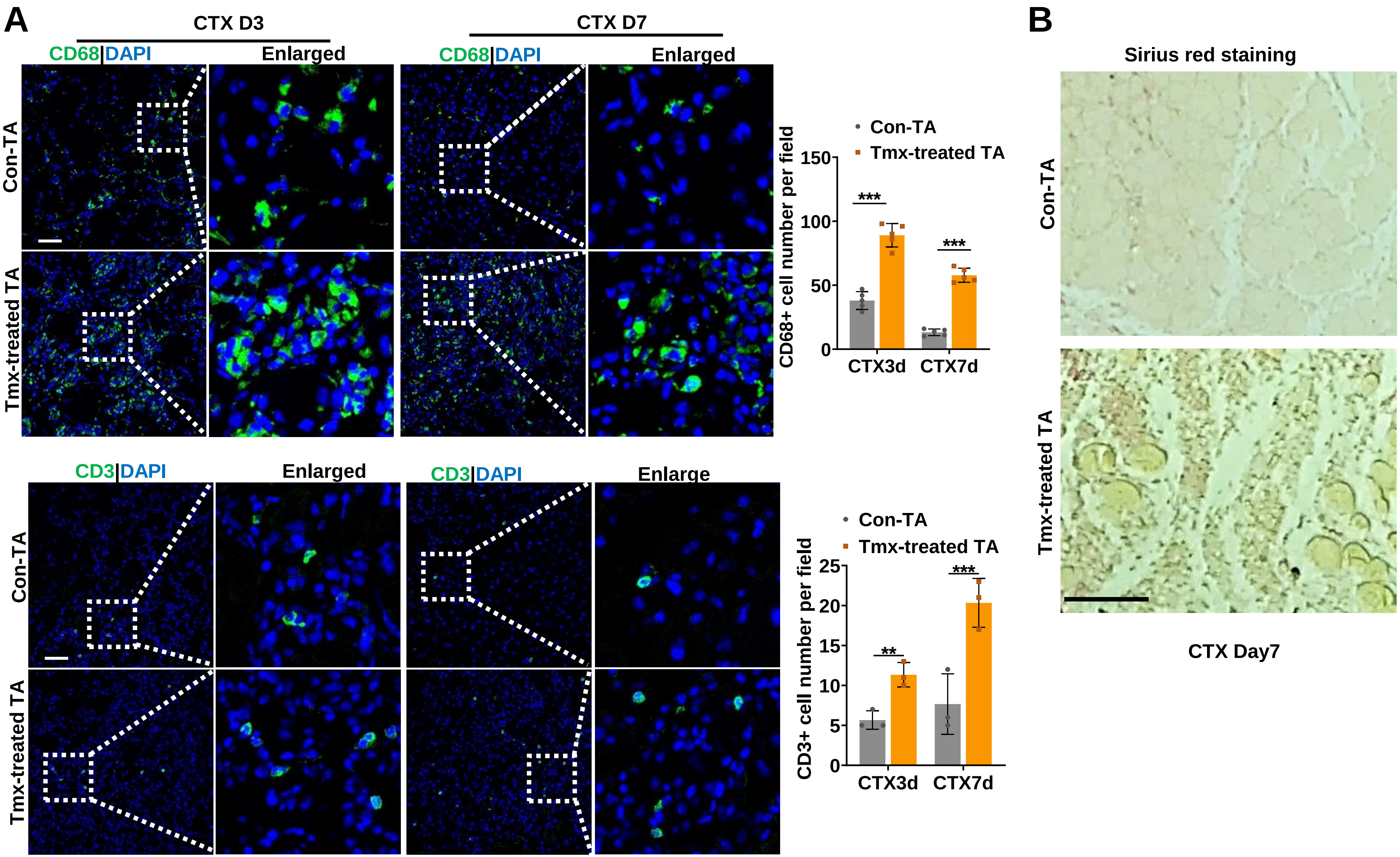

Figure S11. Increased inflammatory response was observed in Tmx-treated TA muscles. (A) Immunostaining of CD68 and CD3 in TA muscle sections from control and Tmx-treated mice on day3 and 7 after injury. Scale bars, 50 μm. The bar graphs on the right shows the quantification of CD68+ cells or CD3+ cells per field (0.18 mm^2) (n=5 per group for CD68 staining and n=3 for CD3 staining). (B) Representative images of Sirius red staining in TA muscle sections from control and Tmx-treated mice on day 7 after injury. Scale bars, 250 μm. Results are Mean ± SD, *P ≤ 0.05, **P ≤ 0.01, ***P ≤ 0.001 (Two-way ANOVA for A, Bonferroni's multiple comparison test).

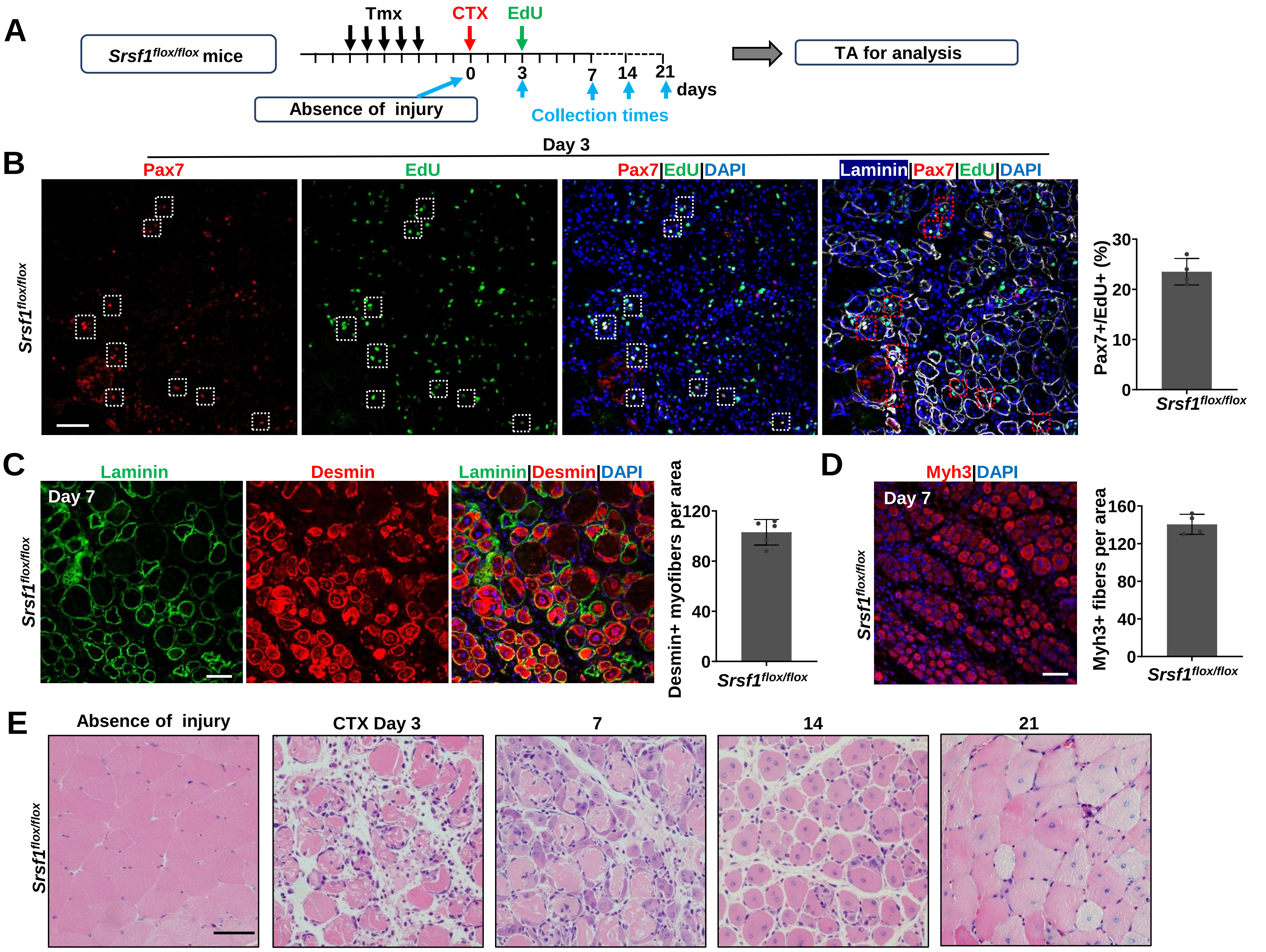

A
Srsf1flox/flox mice
Tmx
CTX
EdU
0
3
7
14
21
days
Absence of injury
Collection times
TA for analysis
B
Day 3
Pax7
EdU
Pax7|EdU|DAPI
Laminin|Pax7|EdU|DAPI
Srsf1flox/flox
Pax7+/EdU+ (%)
30
20
10
0
C
Laminin
Desmin
Laminin|Desmin|DAPI
Day 7
Desmin+ myofibers per area
120
80
40
0
D
Myh3|DAPI
Day 7
Myh3+ fibers per area
160
120
80
40
0
E
Absence of injury
CTX Day 3
7
14
21

Figure S12. *Srsf1$^{flox/flox}$* mice treated with tamoxifen successfully resored muscle regeneration following mucle injury. (A) The experimental strategy involved multiple timelines of interventions, including Tmx injection, CTX injection, EdU injection, and collection times of TA samples, both in the absence of injury and after CTX-induced injury in *Srsf1$^{flox/flox}$* mice. (B) Immunostaining of Pax7 (red) and Laminin (white) staining in TA muscle sections on day 3 after injury. EdU was detected using Alexa-488 labeling (green) and nuclei were stained with DAPI (blue). Dotted boxes indicated merged EdU+ and Pax7+ cells. Scale bars, 50 μm. Quantification of Edu+/Pax7+ cells per area is shown on the right bar graph (n=4 per group). (C) Immunostaining of Laminin (green), Desmin (red) and DAPI (blue) in TA muscle sections on day 7 after CTX injury. Scale bars, 50 μm. The right bar graph shows the quantification of Desmin+ myofibers per field (n=5 per group). (D) Immunostaining of Myh3 (red) in TA muscle sections on day 7 after CTX injury. Scale bars, 50μm. The right bar graph shows the quantification of Myh3+ myofibers per field (n=4 per group). (E) Representative HE images of TA muscles harvested at different time points (absence of injury, 3, 7, 14, and 21 days after CTX injury. Scale bars, 50μm. Results are Mean ± SD.

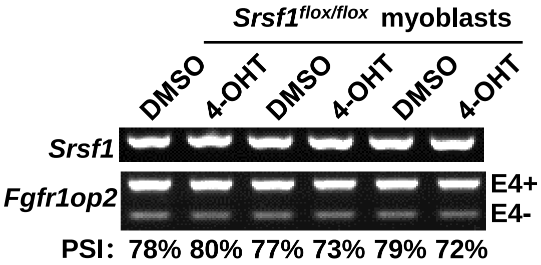

Figure S13. 4-OHT treatment doesn't cause exon 4 skipping of Fgfr1op2 in primary myoblasts from *Srsf1*$^{flox/flox}$ mice. RT-PCR showed mRNA levels of *Srsf1* and two *Fgfr1op2* isoforms in both DMSO-treated and 4-OHT-treated myoblasts. PSI values were presented at the bottom.

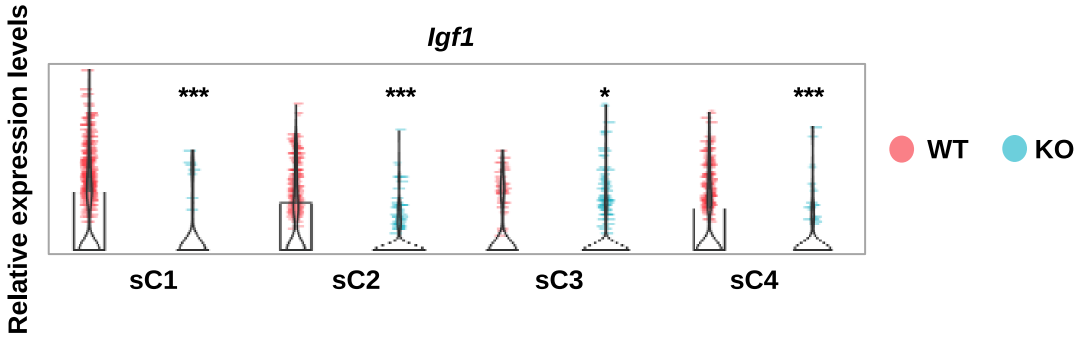

Figure S14. reduced *Igf1* levels in subclusters of KO group. Violin plots demonstrated the expression levels of Igf1 in subclusters between WT and KO groups.

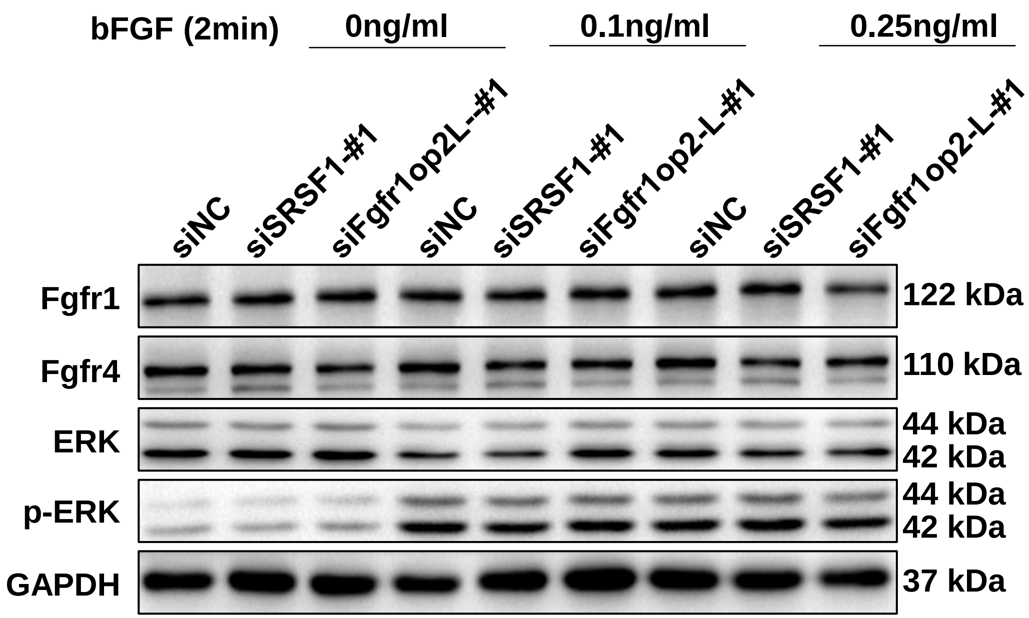

Figure S15. No changes were observed in the Fgf and MAPK pathway following the knockdown of SRSF1 or the Fgfr1op2-L isoform. C2C12 myoblasts were transiently transfected with the indicated siRNA for 48 hours. basic FGF (PeproTech, 100-18B) was added for 2 minutes, and cells were harvested for WB analysis using the indicated antibodies.

Table S1. Primer sequences used for mice genotyping

Gene	Forward (5'–3')	Reverse (5'–3')	Size (bp)
Srsf1$^{flox/flox}$	ACTAATGTGGGAAGAATGGC	AAACTATTGCTCCCATCTGC	400/350bp
Myod1-Cre	GCGGATCCGAATTCGAAGTTCC	TGGGTCTCCAAAGCGACTCC	150bp
Pax7-CreER	GCTGCTGTTGATTACCTGGC	Mut：CAAAAGACGGCAATATGGTG	235bp
		WT：CTGCACTGAGACAGGACCG	419bp

Table S2. Antibodies used in WB and IF

Antibody	Origin	Catalog number	Assay
Beta-actin	Santa Cruz	Cat# sc47778	WB
GAPDH	ABclonal	Cat# A19056	WB
Pax7 Antibody	DSHB	Cat# PAX7-c	IF
Myogenin	Santa Cruz	Cat# sc-576	IF
Myosin heavy chain (MHC) Antibody	Santa Cruz	Cat# sc-20641	IF
SRSF1 Antibody	Millipore	Cat# MABE936	WB
Fgfr1op2 Antibody	Abcam	Cat# ab229119	IF, WB
Pax7 Antibody	Abcam	Cat# ab187339	IF
MyoD Antibody	BD Biosciences	Cat# 554130	IF, WB
Myosin heavy chain (MHC) Antibody	Santa Cruz	Cat# sc32732	IF, WB
Laminin-α2 Antibody	ENZO	Cat# ALX-804-190	IF
Alexa Fluor™ 647 Phalloidin	YEASEN	Cat# 40762ES75	IF
Pericentrin Antibody	Abcam	Cat#ab4448	IF
Alpha-Tubulin Antibody	Santa Cruz	Cat# sc32293	IF
Ki67 Antibody	BD Biosciences	Cat# 550609	IF
Ki67 Antibody	Invitrogen	Cat# PA5-19462	IF
p21 Antibody	Abcam	Cat#ab109199	IF, WB
p53 Antibody	Cell Signaling Technology	Cat# 2524s	WB
Retinoblastoma Protein (pRb) Antibody	BD Biosciences	Cat# 554136	WB
Phospho-Rb (Ser807/811) Antibody	Beyotime	Cat# AR092	WB
CyclinB1 Antibody	Cell Signaling Technology	Cat# 4135	WB
CDK1 Antibody	Beyotime	Cat# AF1516	WB
Aurka Antibody	Abcam	Cat# ab13824	WB
AKT	Cell Signaling Technology	Cat# 4685	WB

P-AKT (S473)	Cell Signaling Technology	Cat# 4070	WB
P-P70S6K	Santa Cruz	Cat# sc8416	WB
FGFR1	ABclonal	Cat# A21219	WB
FGFR4	ABclonal	Cat# A9197	WB
p44/42 MAPK (Erk1/2)	Cell Signaling Technology	Cat# 4695T	WB
Phospho-p44/42 MAPK (Erk1/2)	Cell Signaling Technology	Cat# 4370T	WB
CD68	BioLegend	Cat# 137006	IF
CD3	BioLegend	Cat# 100202	IF
Goat anti-Rabbit IgG (H+L) Cross-Adsorbed Secondary antibody, Alexa Fluor 546	Thermo Fisher Scientific	Cat# A-11035	IF
Goat anti-Rabbit IgG (H+L) Cross-Adsorbed Secondary antibody, Alexa Fluor 488	Thermo Fisher Scientific	Cat# A-11034	IF
Goat anti-Mouse IgG (H+L) Cross-Adsorbed Secondary antibody, Alexa Fluor 546	Thermo Fisher Scientific	Cat# A-11030	IF
Goat anti-Mouse IgG (H+L) Cross-Adsorbed Secondary antibody, Alexa Fluor 488	Thermo Fisher Scientific	Cat# A-32723	IF
Alexa Fluor® 647 AffiniPure Donkey Anti-Rat IgG (H+L)	Jackson ImmunoResearch	Code: 712-605-150	IF

Table S3. Primer sequences used for mRNA expression analysis

**Gene**	**Forward (5'–3')**	**Reverse (5'–3')**	**Size(bp)**
Rplp0	TAAAGACTGGAGACAAGGTGGGAG	AGAAAGCGAGAGTGCAGGGC	166
Srsf1	CGCATCTACGTGGGTAACCT	TAGCCGTCGTAGTCGTAGCC	197
Fgfr1op-L	ATCGTAACAGCTGCGAAGGA	TTCTGATTTGCCTCCAAGTG	100
Kif2c	TGACGATGTGGCTGCAATAAA	TTTGCTTCGGGACCGTTACG	101
Nuf2	TCCCCAGATACAATGTAGCTGA	GCTCTTCGAGAGGTTTTTGCC	86
Spc25	TGTGTTGAAGATGATTGCTGAA	CCTCTTCCTAGCGTACTCTTCC	100
Spdl1	AAAGTGGAATTGGATGAAGCA	AGCCGGAGCTCTTCTGACTT	101
Plk1	TGAAGGGGAACGAAAGAAGA	AATGGACCACACATCCACCT	103
Ndc80	GCCTCTCTATGCAGGAGTTAAGG	CGGTTTGTGTGTACTCAGCTT	107
Aurkb	CAGAAGGAGAACGCCTACCC	GAGAGCAAGCGCAGATGTC	176
Ndufa7	GTTGTGCCTCCCTCAATCAT	TCACTGCCTTCTTCTCAGTGG	100
Ndufaf1	GGAAGGGTACGAGATGTCCA	GGGCTGGATCAGTAAACACA	133
Sdhb	GGAGGGCAAGCAACAGTATC	CTTGTCTCCGTTCCACCAGT	124
Uqcr11	AGAAACTGGATTCCCACAGC	ATGTAAGGCACCCAGTCCAG	101
Cox7a1	GCTCTGGTCCGGTCTTTTAG	CCCGCCTTTCAAGTGTACTG	114
Cox19	GGACCACTTCGGTGAATGTAA	TCTAAAGCCGAGCTTCTCTAGC	166
Atp5d	TGCTTCAGGCGCGTACATAC	CACTTGCTTGACGTTGGCA	128

Atp5h	GCTGGGCGTAAACTTGCTCTA	CAGACAGACTAGCCAACCTGG	136
Cdkn1a	CTTGTCGCTGTCTTGCACTC	AATCTGTCAGGCTGGTCTGC	104
Trp53	GCTCCTCCCCAGCATCTTAT	TGGCTCATAAGGTACCACCA	102
TNF-α	CCGATGGGTTGTACCTTGTC	TGGAAGACTCCTCCCAGGTA	217
IL10	AAGGACCAGCTGGACAACAT	TCTCACCCAGGGAATTCAAA	172
Atf4	GTTTGGGGGCTGAAGAAAG	GCCAAGCCATCATCCATAG	130
Sqstm1	CTGTGGTGGGAACTCGCTAT	AAGGGGTTGGGAAAGATGAG	115
Nfe2l2	CAGTCTTCACTGCCCCTCAT	CCAAACTTGCTCCATGTCCT	101

Table S4. siRNA sequences used for RNA interference

siNC	UUCUCCGAACGUGUCACGUTT
siSRSF1-#1	GAGGCAGGUGAUGUAUGUUTT
siSRSF1-#2	CUGGCAGGACUUAAAGGAUTT
siFgfr1op2-L-#1	GUAACAGCUGCGAAGGAUUTT
siFgfr1op2-L-#2	GAGGCACUUGGAGGCAAAUTT

Cell Culture, siRNA transfection, plasmid construction and transfection

C2C12 cells were cultured at 37°C with 5% CO2 in growth medium (GM) containing DMEM, 10% FBS, and 1% penicillin/streptomycin. Differentiation medium (DM) containing DMEM, 2% horse serum, and 1% penicillin/streptomycin was used to induce differentiation of C2C12 myoblasts. For siRNA transfections, cells were seeded at 10% density and transfected using Lipofectamine RNAiMAX (Invitrogen). The Fgfr1op2-L cDNA was cloned into the pcDNA3.0-HA vector. Co-transfection of siRNA and plasmid was done at a 20% cell density using Lipofectamine 2000 (Invitrogen). The siRNA oligonucleotides, synthesized by Gene Pharma (Shanghai, China), are listed in Table S2.

Primary myoblasts isolation and culture

Primary myoblasts were isolated from P5 *Srsf1*$^{flox/flox}$*;Pax7-CreER* mice and *Srsf1*$^{flox/flox}$ mice by collecting limb skeletal muscles, digesting them, and filtering the resulting suspension. After centrifugation, cells were resuspended in a 20% FBS/F-10 buffer and plated three times to remove adherent cells and enrich myoblasts. The purified myoblasts were cultured on collagen-coated dishes in growth medium containing 20% FBS, and 5ng/ml basic FGF. Srsf1 deletion in vitro was induced by culturing myoblasts with 2uM 4-Hydroxytamoxifen (4-OHT) for 2 days, followed by 4 days without 4-OHT before analysis.

HE, TUNEL, Sirius red staining, Immunostaining and Western blotting

For HE staining, 5 µm paraffin sections followed a standard staining protocol. TUNEL staining was performed on paraffin sections according to the manufacturer's protocol (Promega, catalog no. G3250). For Sirius red staining, paraffin sections were deparaffinized and rehydrated, and then stained with staining solution for 15 minutes. Immunofluorescence (IF) staining was carried out on 6 µm paraffin sections or frozen sections. Paraffin tissues underwent deparaffinization and rehydration, while both types of sections were permeabilized with 0.4% TritonX-100 in PBS for 15 minutes and blocked with 5% NGS (normal goat serum in 0.4% Triton X-100 in PBS) for 1 hour. Primary antibody incubation occurred overnight at 4 °C, followed by 1-hour incubation with secondary antibodies. Cellular immunostaining involved initial fixation with 4%

paraformaldehyde for 15 minutes, permeabilization, primary and secondary antibody staining. IF images were acquired using a LSM880NLO Zeiss microscope and analyzed with ZEN software. Western blotting (WB) followed a standard protocol. Details about the antibodies used in IF and WB can be found in Table S2.

RNA isolation, cDNA synthesis and quantitative real-time PCR (qPCR)

RNA isolation, cDNA synthesis and quantitative real-time PCR followed standard protocols. Total RNA was extracted from mouse tissue samples or C2C12 cells using Trizol Reagent (Invitrogen), while cDNA synthesis employed oligo (dT) priming and M-MLV Reverse Transcriptase. Gene expression was analyzed using standard qPCR reactions with the SYBR Premix Ex Taq kit. Relative gene expression levels were determined via the 2-ΔΔCt method using Rplp0 as a control. Primer sequences for qPCR are listed in Table S3.

RNA-seq data analysis

Diaphragm total RNAs from P1 mice underwent paired-end RNA-seq via Illumina NovaSeq 6000 as per manufacturer guidelines. Raw reads were quality controlled, mapping only high-quality reads to the mouse genome/transcriptome (mm10) using TopHat. Shanghai Personalbio (Shanghai, China) conducted cDNA library construction, sequencing, and transcriptome analysis. Raw datasets are accessible in the SRA database under accession number PRJNA973934.

EdU incorporation assay

The EdU incorporation assay followed the standard protocol. Mice received intraperitoneal EdU injections (100 ug per mouse) six hours before sacrifice to monitor SC proliferation in injured muscle. TA muscles were collected, embedded in O.C.T., and sectioned. EdU incorporation was detected using the Click-iT Edu Kit (Invitrogen) as instructed. In the cell EdU incorporation assay, C2C12 myoblasts or primary myoblasts were incubated with EdU (20 uM) for 2 hours, fixed with 3.7% paraformaldehyde, washed with 3% BSA, and permeabilized with 0.4% Triton X-100 for 15 minutes before EdU detection using the Click-iT Edu Kit.

Cell synchronizations and cell cycle analysis

C2C12 cells were transfected with specific siRNA for 36 hours, then treated with 1mM thymidine for 18 hours. After three PBS washes, cells were shifted to thymidine-free

medium for 10 hours. Subsequently, they were exposed to 1mM thymidine for another 12 hours, washed with PBS, and released into thymidine-free medium. At various time points after release, cells were harvested, fixed with 70% ethanol at -20°C overnight, and stained with propidium iodide (Beyotime Biotechnology). Cell cycle distribution was evaluated using a flow cytometer (Beckman Coulter, CytoFlex LX), and the results were analyzed using FlowJo.

SA-β-gal staining

SA-β-gal staining was performed using the Lysosomal β-Galactosidase Staining Kit. C2C12 cells, after siRNA transfection and 5-day culture, underwent washing, fixation, and staining at pH 6.0.
